# Supplementary material for: Arginine depletion attenuates renal cystogenesis in tuberous sclerosis complex model
Source: Cell Rep Med. 2023 Jun 7;4(6):101073. doi: 10.1016/j.xcrm.2023.101073 (PMC10313931; doi:10.1016/j.xcrm.2023.101073)
Supplement: Document S1. Figures S1–S10 [file mmc1.pdf]

Cell Reports Medicine, Volume *4*

Supplemental information

Arginine depletion attenuates renal cystogenesis in tuberous sclerosis complex model

Athar Amleh, Hadass Pri Chen, Lana Watad, Ifat Abramovich, Bella Agranovich, Eyal Gottlieb, Iddo Z. Ben-Dov, Morris Nechama, and Oded Volovelsky

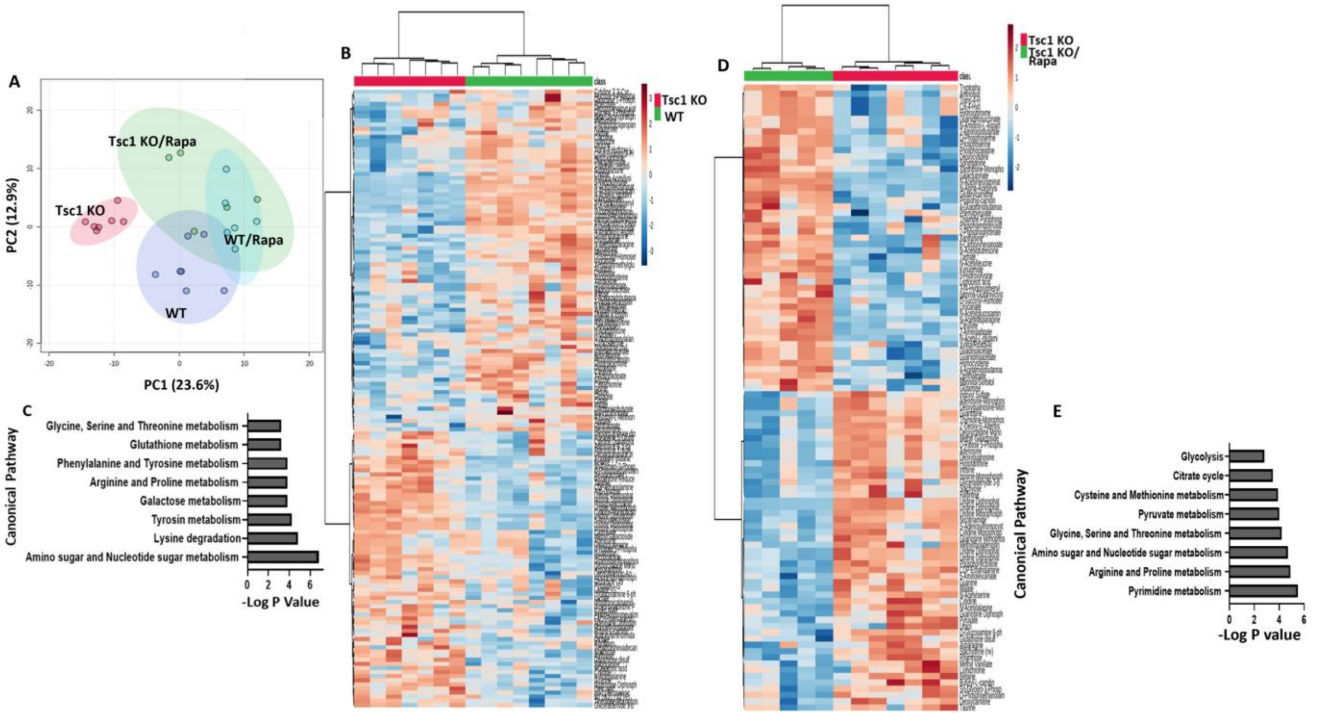

Figure S1: Rapamycin treatment alters kidney metabolic programming in *Tsc1* KO mice, Related to Figure 1.

(A) Principal components analysis plot of WT mice and *Tsc1* KO mice treated with either vehicle or rapamycin during pregnancy. Metabolic analysis of vehicle-treated *Tsc1* KO and WT mice (**B-C**) as well as *Tsc1* KO mice treated with either rapamycin or vehicle (**D-E**). (**B**) and (**D**) show heat maps constructed via hierarchical clustering based on significantly dysregulated metabolites. (**C**) and (**E**) show pathways enriched with the respective dysregulated metabolites in each comparison.

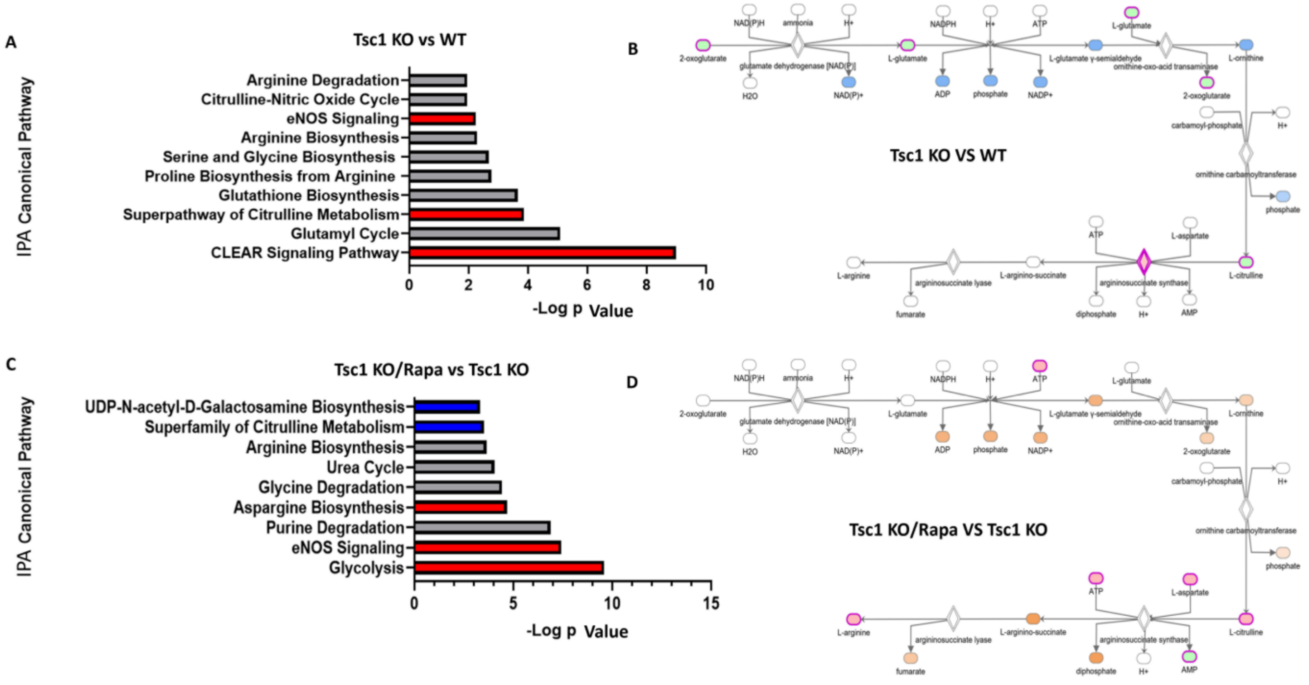

Figure S2: Potential Canonical Pathways and significantly differentially expressed genes and metabolites affected by *Tsc1*K O and treatment with rapamycin, Related to Figure 2.

QIAGEN IPA® of canonical pathways that may be affected (log P-value>1.3) by the significantly differentially expressed genes and metabolites of *Tsc1* KO vs. WT (**A**) and *Tsc1* KO vs. *Tsc1* KO mice treated with rapamycin (**C**). QIAGEN IPA® z-scores larger than 2 or smaller than -2 are considered significantly predicted to be up (red bars) or down (blue bars)- regulated. Gray bars are for canonical pathways with no calculated QIAGEN IPA® z-scores. Scheme of QIAGEN IPA® of arginine Biosynthesis IV canonical pathways for *Tsc1* KO vs. WT (**B**) and *Tsc1* KO treated with rapamycin vs. *Tsc1* KO treated with vehicle (**D**). Experimentally significantly differentially expressed genes and metabolites are highlighted in magenta and colored in pink (upregulated) and green (downregulated). Predicted upregulated and down-regulated genes and metabolites, using the QIAGEN IPA® MAP (Molecule Activity Predictor) tool, are depicted in orange and blue, respectively. Ellipse and rhombus represent chemical and kinase molecules, respectively.

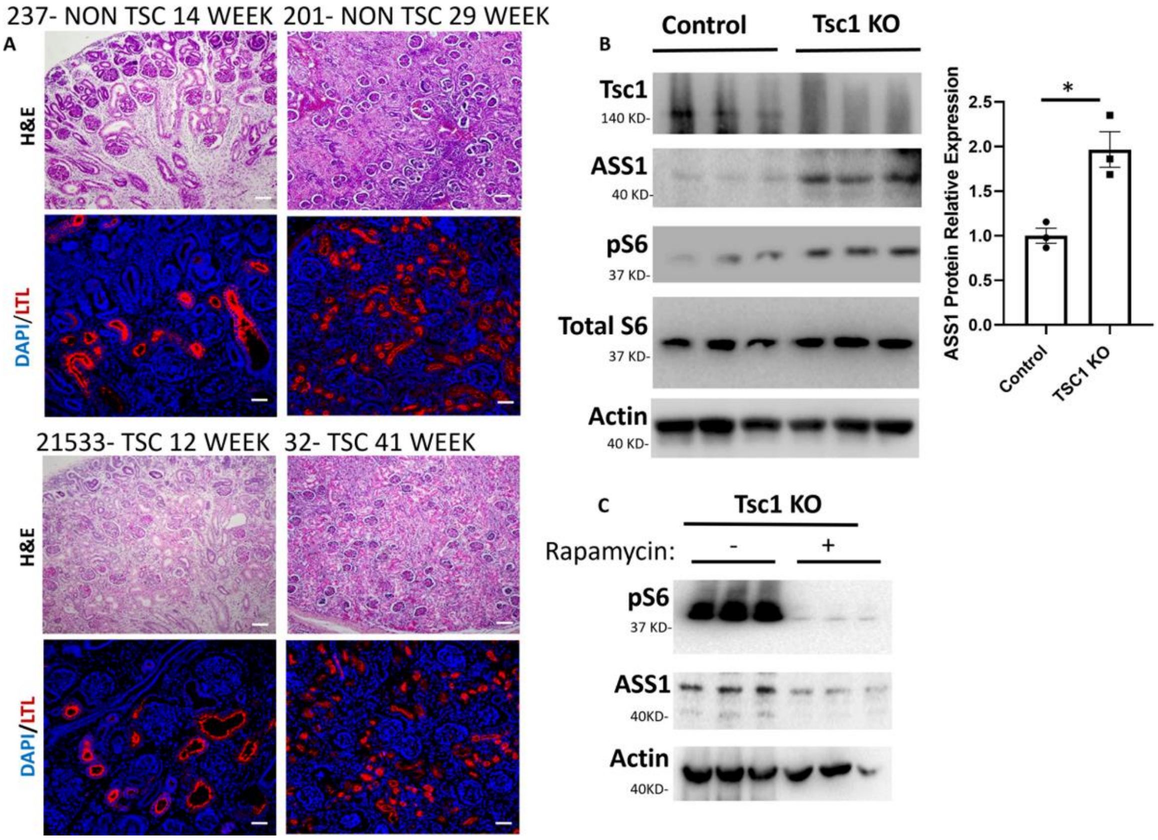

Figure S3: *Tsc1* KO induces ASS1 expression in a mTORC1-dependent manner, Related to Figure 3.

Embryonic kidney sections from human TSC and non-TSC patients in early and late matched pregnancy were H&E stained or immunostained for Lotus Tetragonolobus Lectin (LTL) as a specific marker for PTCs (**A**), showing no difference in the immunostaining. (**B**) Western blot for TSC1, pS6 (a marker for mTORC1 activation), Total S6, Actin, and ASS1 in extracts obtained from control or *Tsc1* KO-induced HK2 cells (n=3 in each group, biological replicates). On the right panel, ASS1 relative protein expression is indicated. *$p < 0.05$. (**C**) Western blot analysis for ASS1, pS6, and actin protein expression in *Tsc1* KO HK2 either incubated with vehicle or 50 nM rapamycin for 24 hours (n=3 in each group, biological replicates).

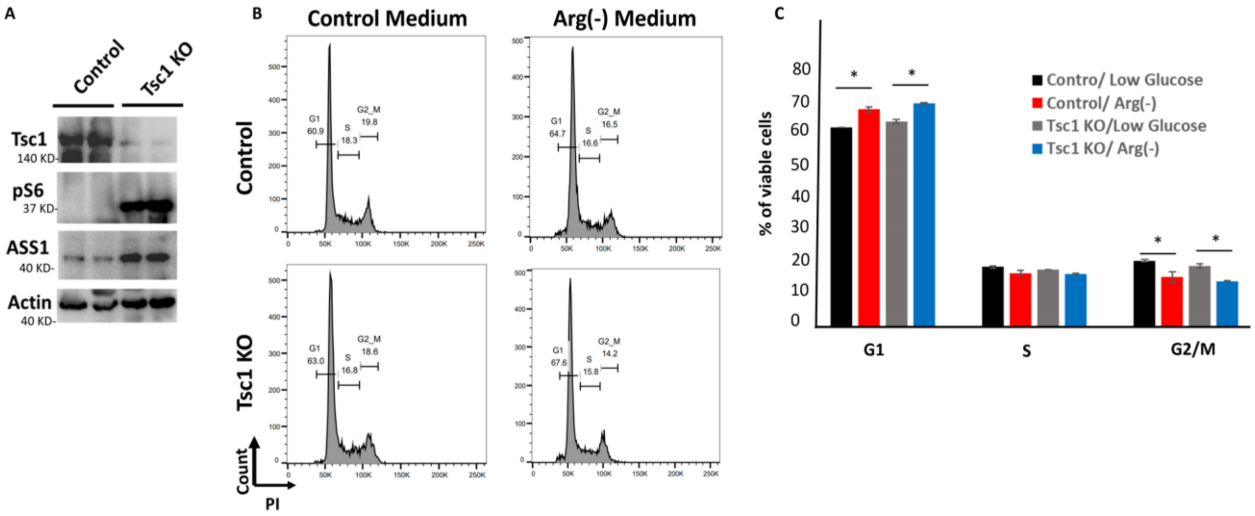

Figure S4: <u>*Tsc1* KO induces ASS1 overexpression in HEK293 cells, Related to Figure 5.</u>

HEK293 cells were cultured in DMEM medium and infected with ASS1/CRISPR or control-containing lentivirus particles. The cells were puromycin and GFP$^+$ based selected. (**A**) Western blot for TSC1, pS6 (a marker for mTORC1 activation), Actin, and ASS1 in extracts obtained from control or *Tsc1* KO-induced HEK293 cells (n=2, biological replicates). (**B**) Control and *Tsc1* KO HEK293 cells were incubated with either control or arginine-free medium for 10 days. Cells were harvested and fixed, and the cell cycle was monitored by propidium iodide flow cytometry-based analysis. (**C**) Quantification as in B (n=3, biological replicates). *p<0.05.

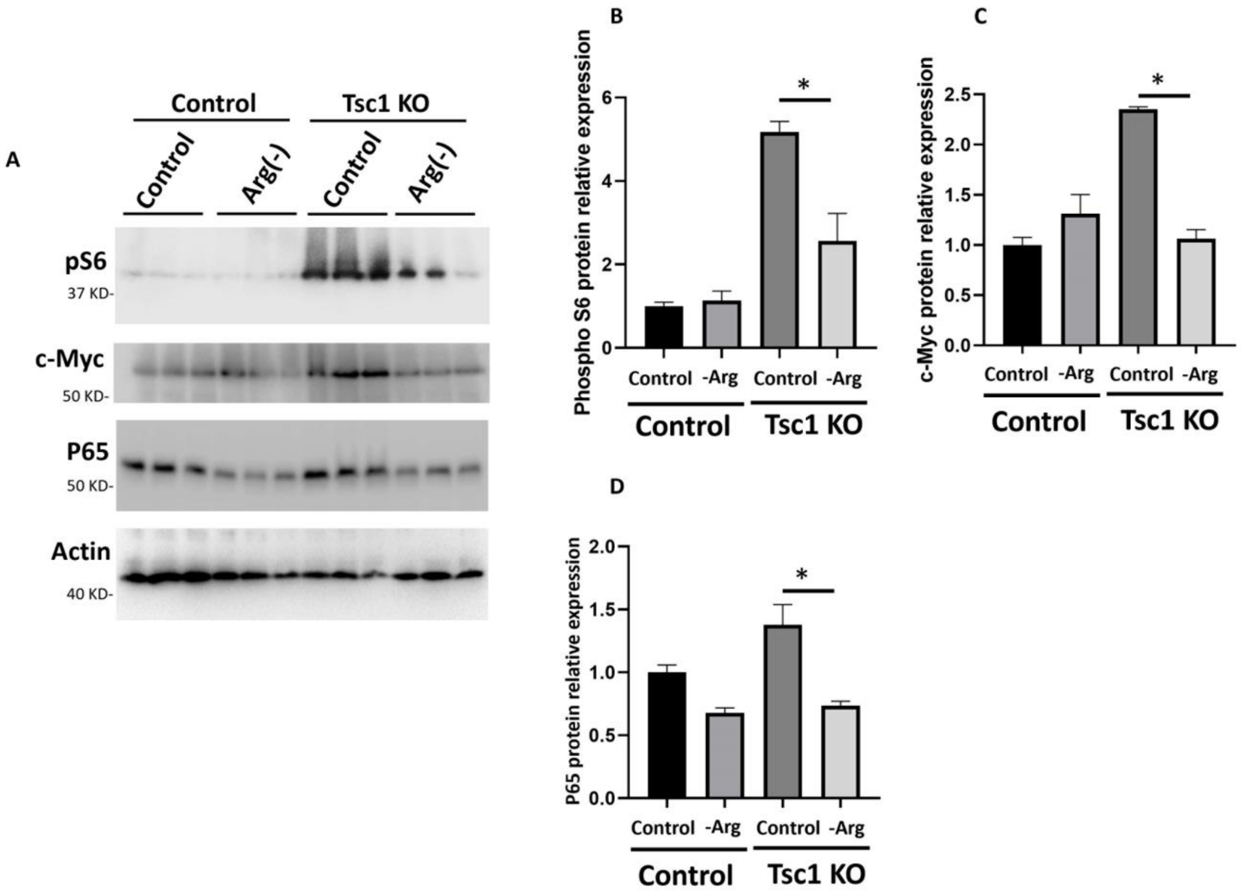

Figure S5: **<u>Arginine depletion *in vitro* attenuates TSC-associated signaling in HEK293 cells, Related to Figure 5.</u>**
Control and *Tsc1* KO HEK293 cells were incubated with either control or arginine-free medium for 10 days. (**A**) Cells were harvested, and the expression pS6, c-Myc, P65, and actin were monitored by western blot analysis, (n=3 in each group, biological replicates). (**B-D**) Quantification of the indicated proteins as in A (n=3, biological replicates). *p<0.05.

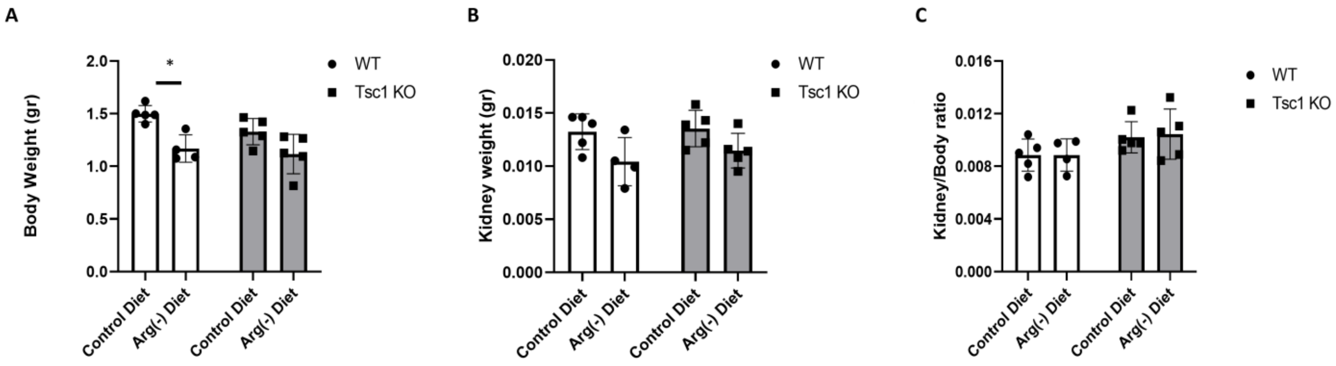

Figure S6: <u>An arginine-deficient diet does not induce kidney and/or body weight changes in newborns, Related to Figure 6.</u>

(**A**) Total body weight of WT and *Tsc1* KO pups from *Tsc1* $^{fl/fl}$ mothers fed with either control or arginine deficient diets at P0 (n=4, three litters, biological replicates) was measured. (**B**) Weight of two kidneys of each pup as in (A) at P0. (**C**) Weight of the two kidneys as in (B) compared with total body weight at P0. *p<0.05.

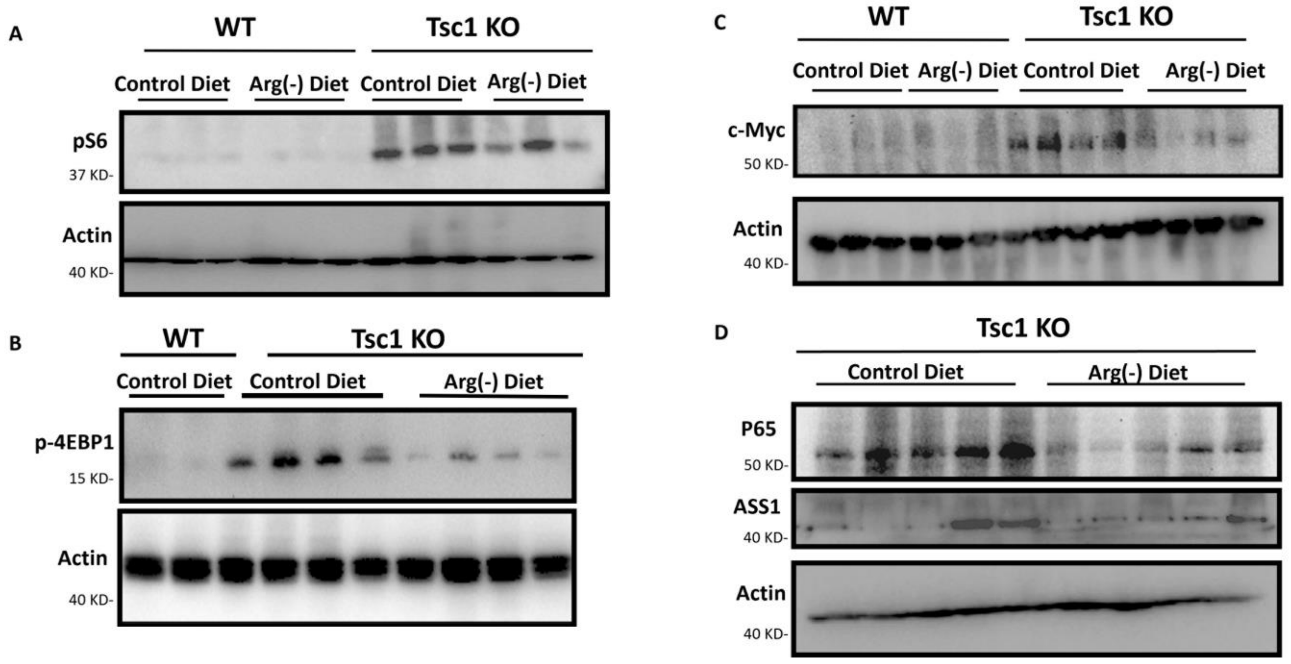

Figure S7: <u>Arginine depletion attenuates mTOR signaling and c-Myc and NF-κB P65 subunit protein expression *in vivo* in *Tsc1* KO kidneys, Related to Figures 6 and 7.</u>

(**A**) Western blot for pS6 and actin protein expression in kidney extracts from WT and *Tsc1* KO pups of *Tsc1* $^{fl/fl}$ mothers fed either a control or an arginine deficient diet, at P0, (n=3 each, biological replicates). (**B**) Western blot for p-4EBP1 and actin protein expression using kidney extracts from WT (n=2 each, biological replicates) and *Tsc1* KO (n= 4 each, biological replicates) pups of *Tsc1* $^{fl/fl}$ mothers fed either control or arginine depleted diets at P0. (**C**) Western blot for c-Myc and β-actin protein expression using kidney extracts from WT (n=3 each, biological replicates) and *Tsc1* KO (n= 4 each, biological replicates) pups of *Tsc1* $^{fl/fl}$ mothers fed either a control or arginine-depleted diet at P0. (**D**) P65, ASS1, and β-actin protein expression using kidney extracts from *Tsc1* KO (n= 5 each, biological replicates) pups of *Tsc1* $^{fl/fl}$ mothers fed either control or arginine-depleted diet, at P0.

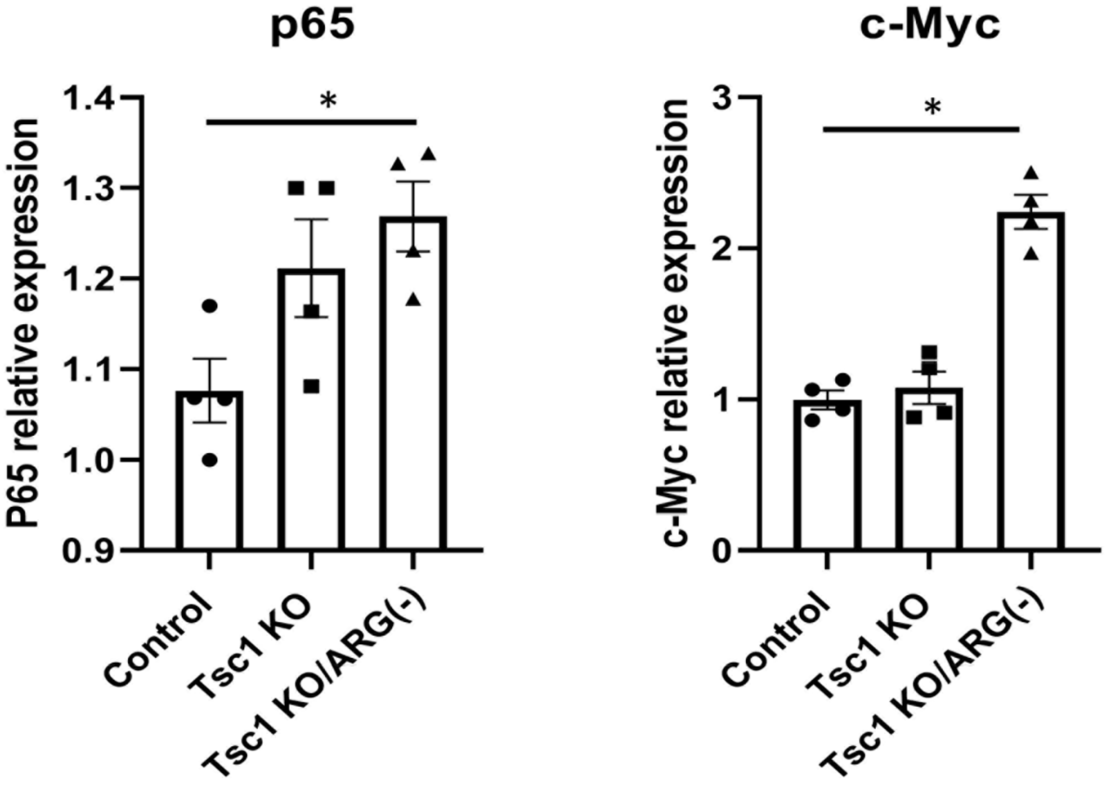

Figure S8: Arginine depletion does not affect c-Myc and P65 gene expression, Related to Figure 7. RNA from WT and *Tsc*1 KO kidneys fed either a control or arginine-depleted diet as indicated were extracted. The relative expression of P65 and c-Myc was monitored and analyzed against β-actin RNA expression by qRT-PCR. WT pups fed a control diet (n = 4, biological replicates), *Tsc1* KO pups fed a control (n = 4, biological replicates), and arginine deficient diet (n = 4, biological replicates), *$p < 0.05$.

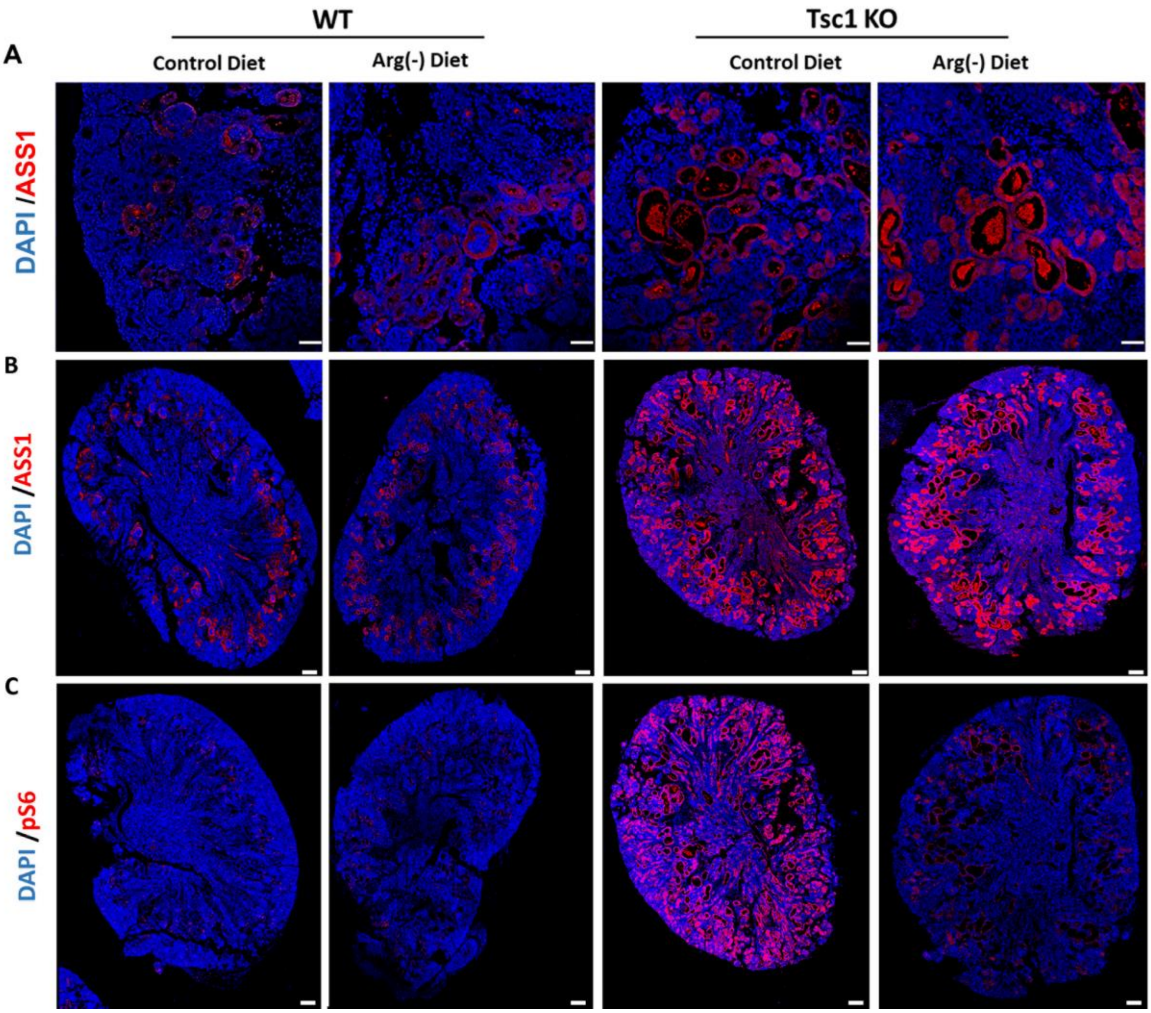

Figure S9: **An arginine-depleted diet does not affect ASS1 expression, Related to Figures 6 and 7.** (**A**) Kidney sections from WT and *Tsc1* KO P0 pups from *Tsc1* $^{fl/fl}$ mothers fed either a control or an arginine-deficient diet were immunostained for ASS1. Scale bar: 50 μm. WT pups were fed a control diet (n = 4, biological replicates) and an arginine-deficient diet (n = 3, biological replicates). *Tsc1* KO pups were fed a control diet (n = 4, biological replicates) and an arginine-deficient diet (n = 4, biological replicates). Sections, as in A, were immunostained for ASS1 (**B**) or pS6 as a marker for mTORC1 activation (**C**). WT pups fed a control diet (n = 3, biological replicates), *Tsc1* KO pups fed a control diet (n = 3, biological replicates), or Arginine depleted diet (n = 3, biological replicates), Scale bar = 500 μm.

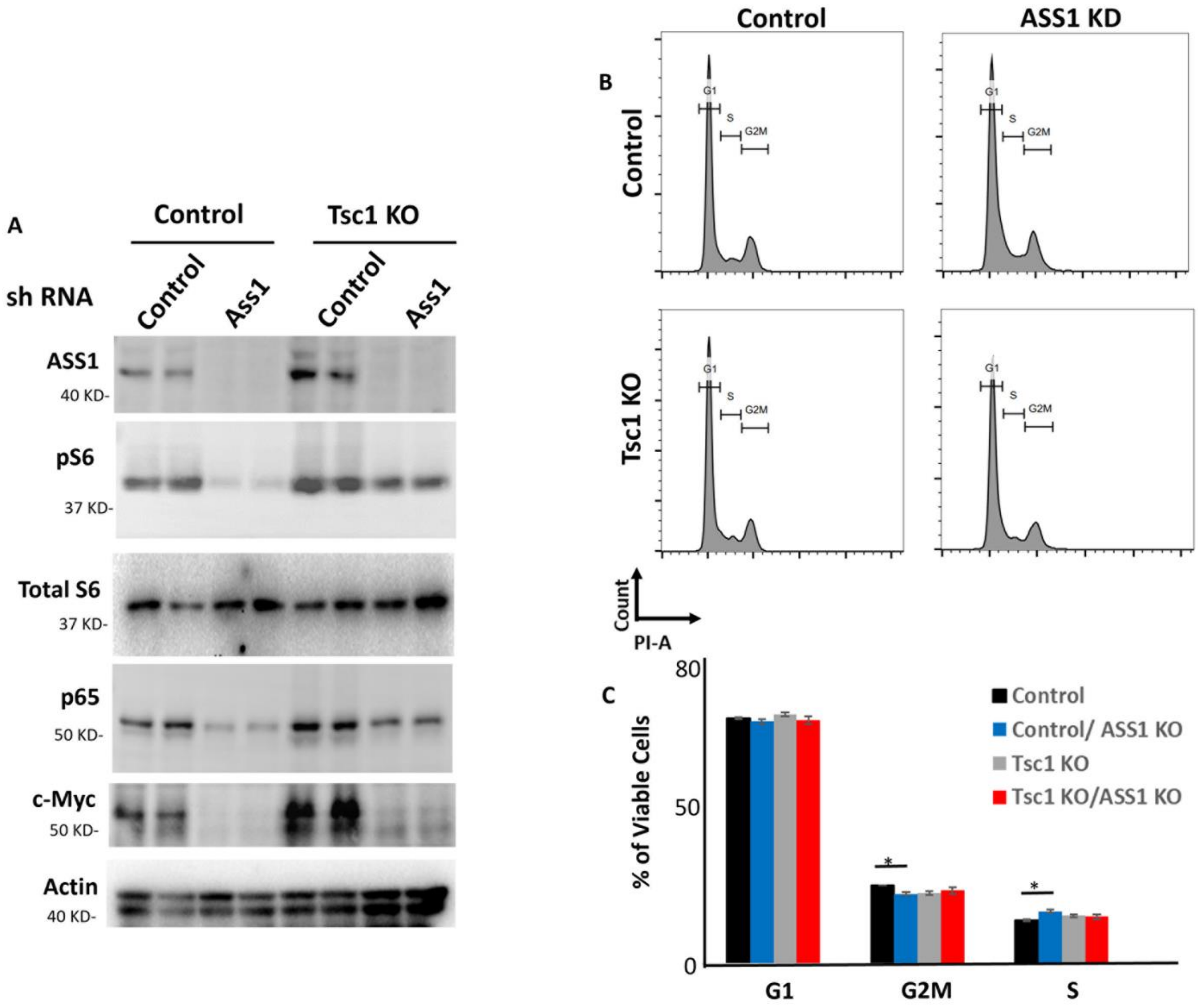

Figure S10: **ASS1 Knock down attenuates TSC associated cell signaling in HK2 cells, Related to Figure 7.**
(**A**) Control and *Tsc1* KO HK2 cells were infected with lentivirus particles containing either control or ASS1-targeted shRNA. After neomycin-based selection, the relative protein expression for ASS1, total and ribosomal protein pS6, P65, c-Myc and actin were evaluated by WB, (n=2, biological replicates). (**B**) Control and *Tsc1* KO HK2 cells, as in A, were harvested and fixed, and the cell cycle was monitored by propidium iodide flow cytometry-based analysis. (**C**) Quantification as in B (n=3, biological replicates). $*p<0.05$.
