# Supplementary material for: Arginine depletion attenuates renal cystogenesis in tuberous sclerosis complex model
Source: Cell Rep Med. 2023 Jun 7;4(6):101073. doi: 10.1016/j.xcrm.2023.101073 (PMC10313931; doi:10.1016/j.xcrm.2023.101073)
Supplement: Document S2. Article plus supplemental information [file mmc8.pdf]

Cell Reports
Medicine

Article

Arginine depletion attenuates renal cystogenesis in tuberous sclerosis complex model

Graphical abstract

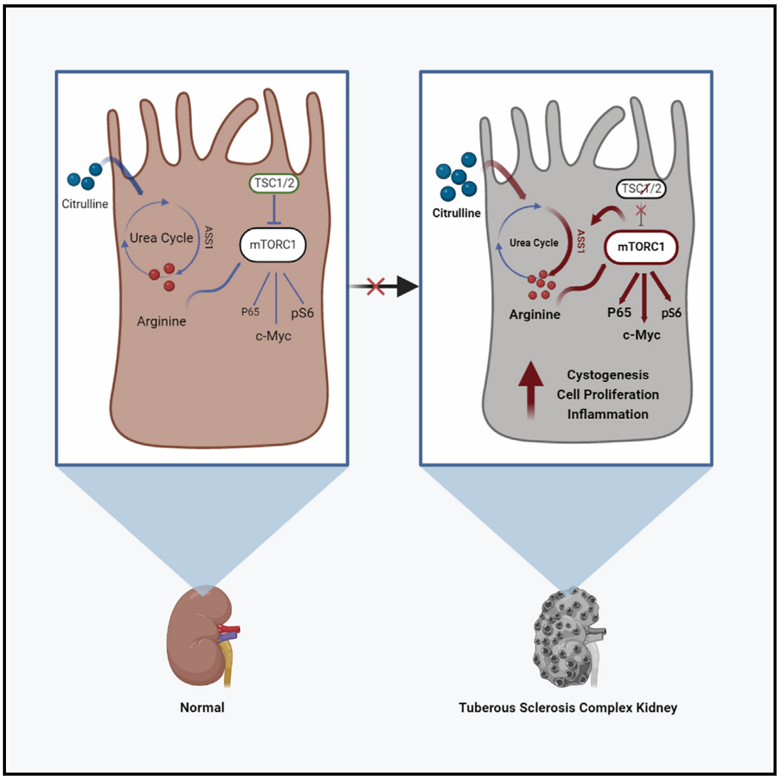

Highlights

- Arginine metabolism is disrupted in TSC disease, leading to renal cystogenesis
- TSC kidneys have high mTOR-dependent argininosuccinate synthetase 1 levels
- Arginine depletion inhibits mTORC1 hyperactivation and cystogenic signaling
- Arginine deprivation might have therapeutic potential for TSC cystic kidney disease

Authors

Athar Amleh, Hadass Pri Chen, Lana Watad, ..., Iddo Z. Ben-Dov, Morris Nechama, Oded Volovelsky

Correspondence

mnehama@gmail.com (M.N.), odedvo@hadassah.org.il (O.V.)

In brief

Amleh et al. reveal that the high metabolic activity of arginine leads to cyst formation in kidney models of tuberous sclerosis complex disease. High mTORC1 activity and signaling pathways in renal cystogenesis can be halted by arginine deprivation in TSC1-deficient cells and mouse kidneys.

Amleh et al., 2023, Cell Reports Medicine *4*, 101073
June 20, 2023 © 2023 The Author(s).
https://doi.org/10.1016/j.xcrm.2023.101073

CellPress

Cell Reports Medicine

CellPress
OPEN ACCESS

Article

Arginine depletion attenuates renal cystogenesis in tuberous sclerosis complex model

Athar Amleh,[1,2] Hadass Pri Chen,[2,3] Lana Watad,[1,2] Ifat Abramovich,[4] Bella Agranovich,[4] Eyal Gottlieb,[4] Iddo Z. Ben-Dov,[3,5] Morris Nechama,[1,2,6,*] and Oded Volovelsky[1,2,6,7,*]
[1]Pediatric Nephrology Unit, Hadassah Medical Center and Faculty of Medicine, Hebrew University of Jerusalem, Jerusalem, Israel
[2]Wohl Institute for Translational Medicine, Hadassah-Hebrew University Medical Center, Jerusalem, Israel
[3]Department of Nephrology, Hadassah Medical Center and Faculty of Medicine, Hebrew University of Jerusalem, Jerusalem, Israel
[4]The Ruth and Bruce Rappaport Faculty of Medicine, Technion – Israel Institute of Technology, Haifa, Israel
[5]Laboratory of Medical Transcriptomics, Department of Nephrology and Hypertension and Internal Medicine B, Hadassah – Hebrew University Medical Center, Jerusalem, Israel
[6]These authors contributed equally
[7]Lead contact
*Correspondence: mnehama@gmail.com (M.N.), odedvo@hadassah.org.il (O.V.)
https://doi.org/10.1016/j.xcrm.2023.101073

SUMMARY

Cystic kidney disease is a leading cause of morbidity in patients with tuberous sclerosis complex (TSC). We characterize the misregulated metabolic pathways using cell lines, a TSC mouse model, and human kidney sections. Our study reveals a substantial perturbation in the arginine biosynthesis pathway in TSC models with overexpression of argininosuccinate synthetase 1 (ASS1). The rise in ASS1 expression is dependent on the mechanistic target of rapamycin complex 1 (mTORC1) activity. Arginine depletion prevents mTORC1 hyperactivation and cell cycle progression and averts cystogenic signaling overexpression of c-Myc and P65. Accordingly, an arginine-depleted diet substantially reduces the TSC cystic load in mice, indicating the potential therapeutic effects of arginine deprivation for the treatment of TSC-associated kidney disease.

INTRODUCTION

Tuberous sclerosis complex (TSC) is a genetic disorder affecting various organs, including the brain, kidney, skin, and heart, with an estimated prevalence of 1:6,000. The disease is caused by inactivating mutations in either the *Tsc1* or *Tsc2* gene, encoding for hamartin and tuberin, respectively.[1–3] TSC1 and TSC2 form a stable complex and function as the GTPase activating factor of the small GTPase Rheb. Stimulation of Rheb-GTP hydrolysis by the TSC1-TSC2 complex inhibits the downstream mechanistic target of rapamycin complex 1 (mTORC1) activity and its targets, including p70 S6 kinase (S6K) and eukaryotic translation-initiation factor 4E-binding protein 1 (4E-BP1), necessary for cell growth, metabolism, and protein synthesis regulation.[4,5] Mutations in *Tsc1/2* genes impair the inhibitory function of the TSC1-TSC2 complex on mTORC1 activity resulting in cell cycle dysregulation and tumorigenesis. Kidney disease is the leading cause of mortality in adult TSC patients and manifests with angiomyolipoma (AML)[6] and cystic kidney disease, identified in the majority of patients.[7] Cystic kidney disease ranges in severity from a single renal cyst to a severe polycystic phenotype, leading to gradual loss of renal parenchyma.[8] Kidney disease is aggravated by the decline in nephron number consequent to multiple surgical procedures for resections and ablations of renal AML of large dimensions.[9] As a result, TSC patients are exposed to CKD complications earlier than the general population, with about 40% of TSC patients developing advanced CKD.[10–14] Renal cystogenesis is attributed to abnormal growth and function of renal tubular cells, but the molecular and metabolic mechanisms underlying TSC-associated cystic kidney disease are not well characterized, and effective therapies are still obscure.

mTORC1 and inflammation have a central role in the pathogenesis of TSC cystic kidney disease. Inactivating *Tsc1* mutations in nephron progenitor cells (NPCs) in mice increased cell proliferation and resulted in severe damage to renal proximal tubule cells (PTCs), starting as early as embryonic day 15.5 (E15.5) with a lethal cystic phenotype at E17.5. Furthermore, this effect was linked to enhanced c-*Myc* expression and increased inflammation, mainly macrophage infiltration, contributing to cyst formation in TSC. Rapamycin or dexamethasone treatment during pregnancy alleviated cystic kidney disease by inhibiting the mTORC1 pathway and the inflammatory response.[15] Other mechanisms associated with TSC cyst development were also proposed. *Tsc2* deletion accelerated extracellular vesicle (EV) production in the damaged cells with a distinct protein reservoir involved in diverse biological processes such as cellular proliferation, stress response, and metabolic pathways. The EVs signal to recipient cells to maintain tissue repair and cellular proliferation, thus contributing to TSC-associated cystogenesis.[9,16]

The limited response of TSC cystic kidney disease to mTOR inhibitors raises the possibility of mTORC1-independent cellular effects through additional cellular pathways. *Tsc1* acts with FNIP1/2 as a co-chaperone to regulate Hsp90 chaperone activity by decelerating its ATPase activity which is essential to Hsp90

© 2023 The Author(s).
This is an open access article under the CC BY license (http://creativecommons.org/licenses/by/4.0/).

function.[17–20] Moreover, *Tsc1* was shown to control tight junction formation to create and maintain the epithelial barrier by mTORC1-independent pathways.[21] *Tsc1* hemizygous deletion in NPCs had mTOR-independent effects on nephrogenesis.[22] *Tsc2* deletion affected prostaglandin production, NOTCH activity, and *VEGF* gene expression in a mTORC1-independent mechanism.[23–25]

Significant metabolic cellular changes have been identified in renal cystic kidney diseases such as autosomal-dominant polycystic kidney disease (ADPKD).[26–30] The role of metabolic changes in TSC has not been extensively examined in TSC cystic kidney disease. However, it can be assumed that mTORC1 hyperactivation causes extensive metabolic reprogramming. mTORC1 is a major regulator of cell metabolism and is controlled by cell environment and nutrient availability. Cells with high mTORC1 activity have extensive metabolic rewiring, including a Warburg-like switch to aerobic glycolysis, enhanced glucose flux through the pentose phosphate pathway, and glutamine addiction.[31] The perturbations in these metabolic pathways sustain the high energy and metabolite demand, thus supporting cellular proliferation and protein synthesis. Indeed, PTCs obtained from TSC mouse kidneys are characterized by significant perturbation in expressions of genes associated with major metabolic pathways such as glucose metabolism, oxidative phosphorylation, the tricarboxylic acid (TCA) cycle, and lipid metabolism.[15]

This study aims to identify the dysregulated metabolic pathways that drive the cystogenic process in a TSC kidney model. Moreover, we aimed to distinguish between the metabolic pathways governed by mTORC1 signaling and mTORC1-independent pathways. We identified perturbation in several key metabolic pathways using the metabolomic analysis of *Tsc1* knockout (KO) mice whole kidneys, specifically in PTCs. Furthermore, we detected changes in arginine metabolism and showed they have a pivotal role in the pathogenesis of TSC kidney disease associated with overexpression of ASS1, a rate-limiting enzyme in the arginine biosynthetic pathway in a mouse model and human TSC kidneys. Accordingly, arginine depletion reduced cell proliferation, mTORC1 activity, and TSC-associated cell signaling, both *in vitro* and *in vivo*. In addition, arginine depletion substantially reduced the cystic load in the TSC mouse model. These results suggest that arginine metabolism plays a critical role in TSC-associated cystogenesis, indicating that targeting this pathway may hold promise as a potential therapeutic strategy for TSC disease.

RESULTS

Rewiring of the metabolic activity in a TSC kidney model

To assess perturbations in major metabolic pathways of *Tsc1* KO kidneys, their association with the cystogenic process, and whether they are governed by mTORC1-dependent or mTORC1-independent pathways, we used transgenic mice with complete *Tsc1* deletion in Six2^{+} NPCs differentiating into the majority of nephron components.[32] We previously showed that these mice have a postnatal lethal phenotype with mTORC1 hyperactivation in PTCs throughout pregnancy and a cystic phenotype visible at E17.5.[15] *Tsc1*$^{fl/fl}$ female mice were mated with *Six2 Cre*$^{tg/+}$ *Tsc1*$^{fl/+}$ males to generate *Six2 Cre*$^{tg/+}$ *Tsc1*$^{fl/fl}$ pups with NPC-specific *Tsc1* deletion (25% of offspring, herein *Tsc1* KO). Pregnant *Tsc1*$^{fl/fl}$ females were injected with vehicle or rapamycin, a potent and specific inhibitor of mTORC1, at E12.5, E14.5, and E16.5, as before.[15] At postnatal day 0 (P0), the kidneys of wild-type (WT) (*Tsc1*$^{fl/fl}$) and *Tsc1* KO mice treated with either rapamycin or vehicle were excised, and metabolites were extracted and analyzed by hybrid triple quadrupole mass spectrometry. Overall, 8 WT and 7 *Tsc1* KO kidneys treated with vehicle and 6 WT and 5 *Tsc1* KO kidneys treated with rapamycin were collected. A total of 298 metabolites were identified (Table S1). Principal-component analysis (PCA) distinguished among the 4 subgroups of kidneys, with the major difference noticed between *Tsc1* KO and WT kidneys. Moreover, the PCA indicated that *Tsc1* KO kidneys treated with rapamycin more closely resembled WT kidneys treated with vehicle (Figure S1A).

To identify alterations in metabolite levels and the metabolic pathways presumably affected by *Tsc1* deletion, we compared metabolite intensity between kidneys from WT and *Tsc1* KO mice treated with vehicle. Overall, 100 metabolites were identified, showing significant differences between the two groups (Figure S1B; Table S2). Furthermore, pathway analysis indicated that the altered metabolites are the products of different metabolic pathways, including amino-sugar and nucleotide-sugar metabolism and amino acid metabolism, such as the metabolism of lysine, tyrosine, and arginine (Figure S1C).

Rapamycin reverses the metabolic profiling in TSC kidney mouse model

We have previously shown that mTORC1 inhibition during pregnancy alleviates cystic kidney disease in *Tsc1* KO offspring by inhibiting the mTORC1 pathway and reducing the inflammatory response.[15] Therefore, we examined the effect of mTORC1 inhibition during pregnancy on the TSC metabolic activity. To this end, we compared metabolite levels between kidneys obtained from *Tsc1* KO pups with or without rapamycin treatment during pregnancy. Overall, 108 metabolites were identified, showing significant differences between the two groups (Figure S1D; Table S3). Pathway analysis indicated that the altered metabolites are associated with different metabolic pathways, including pyrimidine metabolism, amino-sugar, and nucleotide-sugar metabolism, and amino acid metabolism of arginine, glycine, serine, and threonine (Figure S1E).

Next, we focused on identifying specific metabolites and metabolic pathways that were dysregulated in *Tsc1* KO mice compared with WT mice and reversed to the WT kidney profile upon rapamycin treatment. As mentioned above, PCA indicated clear segregation of the sample groups. Strikingly, kidneys obtained from *Tsc1* KO mice treated with rapamycin more closely resembled WT kidneys (Figure 1A). In addition, we noted a general inverse relationship between the *Tsc1* KO effects on metabolite levels and the rapamycin effects on these metabolites in *Tsc1* KO kidneys (Figure 1B). Overall, we identified 58 metabolites that were significantly cross-regulated (Table S4), 34 of which were upregulated in kidneys from *Tsc1* KO mice compared with WT mice and conversely down-regulated upon rapamycin treatment of *Tsc1* KO mice. An additional 24

CellPress
OPEN ACCESS

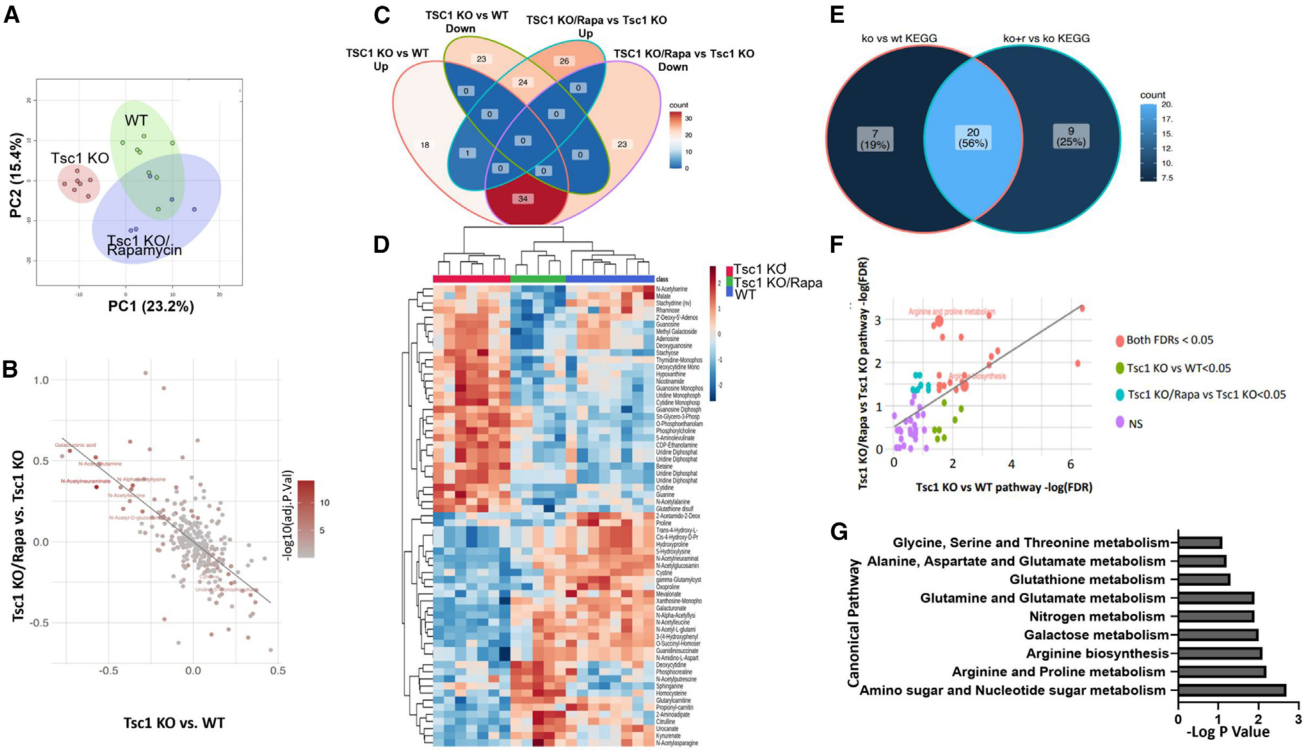

Figure 1. Rapamycin treatment reverses the metabolic profiling of *Tsc1* KO kidneys

(A) Principal-component analysis plot of WT mice and *Tsc1* KO mice with or without rapamycin treatment during pregnancy. Each dot represents an independent biological sample.

(B) Scatterplot (ggplot2) showing log2 fold changes between the change in metabolites levels in *Tsc1* KO and WT (x axis) and with and without rapamycin in *Tsc1* KO mice (y axis), implying a reversal of mutation effects by rapamycin.

(C) Venn diagram (ggVennDiagram) showing the overlap in dysregulated (significantly upregulated or downregulated) metabolites across the two pairwise comparisons.

(D) Heatmap and dendrogram generated via hierarchical clustering of samples and metabolites that were significantly altered in at least 1 of the comparisons depicted in (C).

(E) Venn diagram showing the overlap of metabolic pathways enriched with significantly altered metabolites in either of the depicted comparisons (KO, *Tsc1* KO; KO+r, *Tsc1* KO treated by rapamycin).

(F) Scatterplot showing a correlation of the negative $\log_{10}$ FDR (false detection rate) of pathways affected by the experimental interventions (as in E).

(G) Representative pathways enriched with significantly altered metabolites.

See also Figure S1.

metabolites showed significant downregulation in kidneys obtained from *Tsc1* KO mice compared with WT mice but were upregulated upon rapamycin treatment of *Tsc1* KO mice (Figures 1C and 1D).

Pathway analysis on the basis of the metabolites showing a difference between kidneys from *Tsc1* KO and WT mice indicated that these metabolites are involved in 27 different metabolic pathways. Twenty-nine different metabolic pathways were identified when metabolites showing differences between kidneys from *Tsc1* KO with or without rapamycin were analyzed. The vast majority of these pathways, 20 metabolic pathways, were jointly enriched in the two comparisons (Figures 1E and 1F), indicating that most *Tsc1* KO-affected pathways respond to rapamycin. Nucleotide-sugar metabolism, arginine and proline metabolism, and arginine biosynthesis pathways were the top enriched and shared by the two comparisons (Figures 1F and 1G).

The metabolic shift in TSC proximal tubular cells

The PTCs have high metabolic activity as most water, solutes, glucose, and amino acid reabsorption occur in this nephron segment. Thus, a shift in metabolic activity and nutrient availability may substantially affect the structure and function of the proximal tubules. Indeed, we have previously shown that *Tsc1* deletion in NPCs leads to severe damage to PTCs manifested in swollen cellular appearance with an occluded tubular lumen and deranged mitochondrial structure.[15,22] In addition, PTCs are the primary source of cells responsible for TSC renal cyst development in the *Tsc1* KO mice.[15,22] Therefore, we aimed to identify the specific metabolic shift in PTCs of *Tsc1* KO mice and the effect of rapamycin in these cells. For that purpose, *Tsc1*$^{fl/fl}$ female mice were mated with *Six2 Cre*$^{tg/+}$ *Tsc1*$^{fl/+}$ males and treated with either rapamycin or vehicle, as before.[15] At P0, the kidneys were excised, and PTCs were fluorescence-activated cell sorting (FACS)-based sorted using prominin-1 antibody, a selective PTC marker.[15] The

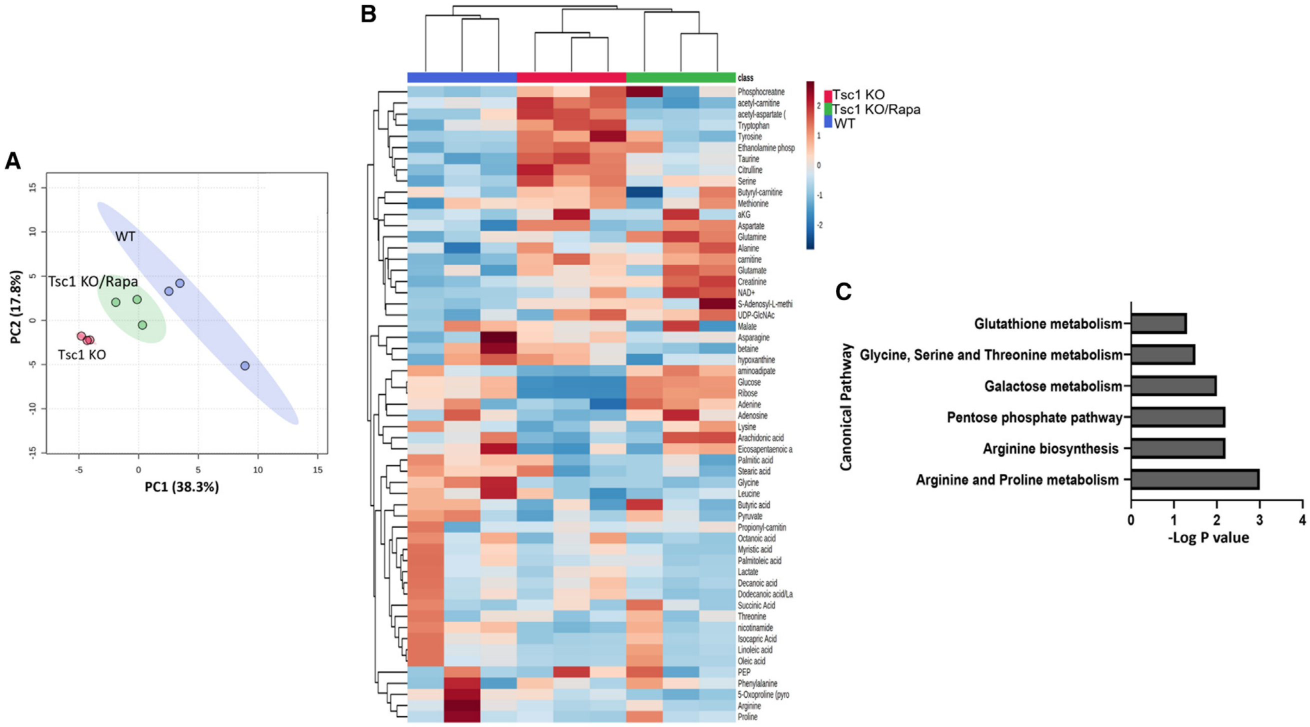

Figure 2. Rapamycin treatment reverses the metabolic profiling of *Tsc1* KO PTCs through a direct effect on the arginine metabolism pathway
(A) Principal-component analysis plot showing WT mice, *Tsc1* KO mice, and *Tsc1* KO mice treated with rapamycin. Each dot represents an independent biological samples.
(B) Heatmap and dendrogram generated via hierarchical clustering of samples and all (58) detected metabolites.
(C) Pathways enriched with significantly altered metabolites. The metabolite concentration tables of the selected groups were analyzed by using MetaboAnalyst 4.0 (https://www.metaboanalyst.ca), applying no filtering, quantile normalization, log transformation, and no scaling.
See also Figure S2.

metabolites were extracted and analyzed by hybrid triple quadrupole mass spectrometry as above. Overall, 57 metabolites were identified (Table S5), with 25 metabolites showing a pattern of dysregulation in PTCs obtained from *Tsc1* KO mice and reversal toward the PTCs obtained from WT kidneys upon rapamycin treatment of *Tsc1* KO mice (Table S6). These changes were associated with various metabolic pathways. The most substantial perturbations were identified in arginine and proline metabolism and arginine biosynthesis pathways (Figures 2A–2C).

To further investigate metabolic pathways altered in TSC, the previously identified differentially expressed genes[15] and the set of significantly altered metabolites identified in this study (Figure S1) were subjected to Ingenuity Pathway Analysis (IPA; Qiagen) gene-metabolite expression analysis. This joint IPA revealed significant perturbations in several metabolic pathways such as the CLEAR signaling pathway, glutamyl cycle, citrulline metabolism, and amino acids such as proline, arginine, serine, and glycine biosynthesis pathways (Figures S2A and S2B). Furthermore, an additional gene-metabolite IPA expression analysis comparing PTC gene expression and metabolic profiling obtained from *Tsc1* KO mice with and without rapamycin indicated a significant perturbation in metabolic pathways such as glycolysis, endothelial nitric oxide synthase (eNOS) signaling, urea cycle, and arginine biosynthesis pathway (Figures S2C and S2D). Of note, some of the metabolic pathways identified, such as citrulline metabolism and eNOS signaling, are linked with the arginine biosynthesis pathway and were predicted to be enriched in *Tsc1* KO kidneys according to the integrated IPA.

High ASS1 expression in the TSC mice model and human samples

Our metabolomic assays presented above anticipated substantial changes in arginine metabolism in *Tsc1* KO kidneys affected by rapamycin, and upregulation of *Ass1* expression in PTCs obtained from *Tsc1* KO mice compared with WT PTCs was depicted by our gene expression analysis (Figure S2). Arginine is a conditionally essential amino acid involved in diverse biological processes such as amino acid and NO production, host immune responses, and cell signaling.[33–40] The *de novo* biosynthetic pathway of arginine involves the conversion of citrulline to arginine and is catalyzed by ASS1 and argininosuccinate lyase (ASL). Specifically, ASS1 catalyzes the condensation of citrulline and aspartate to form argininosuccinate, the immediate precursor of arginine synthesis. ASS1, a rate-limiting enzyme in urea synthesis, is now recognized as a ubiquitous enzyme in mammalian tissues with specific expression and localization in different tissues depending on the specific arginine needs of the tissue.[40] Interestingly, ASS1 overexpression in TSC human organoids resembling AMLs was previously reported.[41]

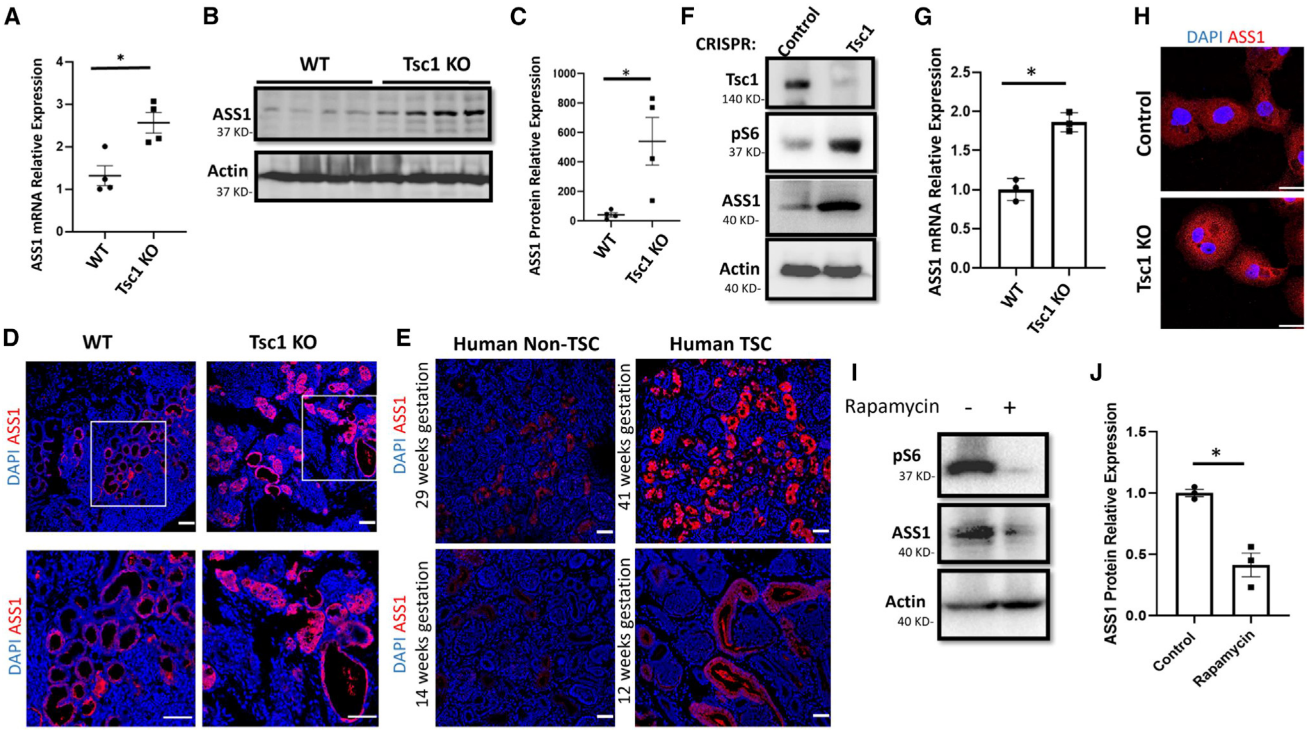

Figure 3. *Tsc1* deletion induces ASS1 overexpression in mTOR-dependent pathway

(A) RNA was extracted from WT, and *Tsc1* KO kidneys at P0, and the relative ASS1 expression was quantified, indicating higher ASS1 expression in the kidneys of *Tsc1* KO mice compared with WT kidneys, $^*p < 0.05$ (n = 4 biological replicates).
(B) Western blot for ASS1 and control GAPDH in control, and *Tsc1* KO mice homogenized kidneys.
(C) Quantification of the western blot as in (B). $^*p < 0.05$ (n = 4 biological replicates).
(D) Renal sections of WT and *Tsc1* KO mice (P0) were stained with anti-ASS1. Scale bar: 50 μm. (n = 3 biological replicates).
(E) Human renal sections of miscarriage fetuses at different embryonic stages due to TSC or non-related causes as indicated, stained with ASS1.
(F) Western blot for TSC1, pS6 (a marker for mTORC1 activation), β-actin, and ASS1 in extracts obtained from control or *Tsc1* KO HK2 cells (n = 3 biological replicates).
(G) Relative ASS1 gene expression using RNA obtained from control and *Tsc1* KO HK2 cells, $^*p < 0.05$ (n = 3 biological replicates).
(H) ASS1 immunostaining of control and *Tsc1* KO HK2 cells (scale bar: 50 μm; n = 3 biological replicates).
(I) Western blot analysis for ASS1, pS6, and β-actin protein expression in *Tsc1* KO HK2 treated with either vehicle or 50 nM rapamycin for 24 h (n = 3 biological replicates).
(J) Quantification for ASS1 relative protein expression as in (I), $^*p < 0.05$ (n = 3 biological replicates).
See also Figure S3.

The increase in ASS1 gene expression and protein levels was validated by qRT-PCR and western blotting (WB), showing an increase in ASS1 expression in *Tsc1* KO kidneys (Figures 3A–3C). Moreover, ASS1 immunostaining of mouse kidney samples indicated high expression in *Tsc1* KO kidneys, specifically in cyst-lining epithelial cells (Figure 3D). Immunostaining of ASS1 in embryonic kidney sections from human TSC sections also demonstrated high levels of ASS1 expression in TSC kidneys compared with matched control embryonic kidneys in early and late pregnancy (Figure 3E). The same human embryonic kidney sections showed no difference in immunostaining with other markers, such as lotus tetragonolobus lectin (LTL), a specific marker for PTCs (Figure S3A), indicating the specificity of ASS1 immunostaining.

The rise in ASS1 levels in proximal tubular cells is mediated by mTORC1 activation

We used a human proximal tubular cell line (HK2 cells) to study the interaction between mTORC1 and ASS1 expression. *Tsc1* was knocked out in HK2 cells using CRISPR-Cas9 containing lentiviral particles. Indeed, *Tsc1* KO increased mTORC1 activity as observed by high pS6 ribosomal protein levels. As observed in the mouse and human models, *TSC1* KO was associated with high ASS1 levels, measured by qRT-PCR, WB, and immunostaining, with no effect on ASS1 cellular localization (Figures 3F–3H and S3B). These results were further reinforced in HEK293 cells, demonstrating that *Tsc1* KO in these cells elevated ASS1 protein expression (Figure S4A). To examine whether ASS1 upregulation is secondary to mTORC1 hyperactivation, *Tsc1* KO HK2 cells were incubated with rapamycin. Rapamycin significantly inhibited mTORC1 activation and ASS1 expression, indicating that ASS1 expression is regulated by the mTORC1 pathway (Figures 3I, 3J, and S3C).

For further reinforcement, ASS1 expression was evaluated in kidney sections obtained from vehicle-treated WT and *Tsc1* KO, as well as *Tsc1* KO mice treated with rapamycin as before.[15] pS6 and ASS1 immunostaining was enhanced in *Tsc1* KO

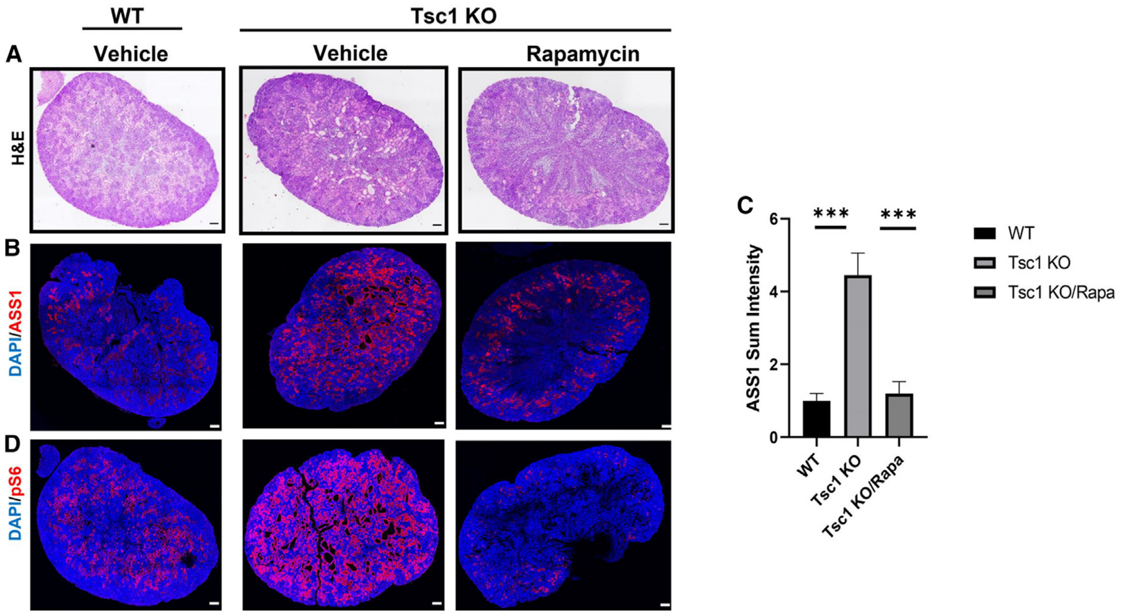

Figure 4. Rapamycin treatment *in vivo* alleviates TSC-associated cyst development and inhibits ASS1 expression in a mTORC1-dependent manner

(A and B) Kidney sections of WT and *Tsc1* KO mice treated with either vehicle or rapamycin, as indicated, were H&E stained (A) or immunostained for ASS1 (B) or pS6 as a marker for mTORC1 activation (D). WT pups treated with vehicle (n = 3 biological replicates), *Tsc1* KO pups treated with vehicle (n = 3 biological replicates), or rapamycin (n = 3 biological replicates). Scale bar = 500 μm.

(C) Relative quantification of sum fluorescence intensity as in (B). $^{*}p < 0.05$.

kidneys compared with WT kidneys and both pS6 and ASS1 immunostaining were diminished upon rapamycin treatment, pointing to mTORC1-dependent ASS1 regulation (Figures 4A–4D). Altogether, our results indicate major perturbations in the arginine biosynthesis pathway, together with mTORC1-dependent overexpression of the rate-limiting enzyme ASS1.

Arginine deprivation impairs the proximal tubular cell cycle and TSC-associated protein expression in *Tsc*1 KO HK2 cells

Next, we studied the effect of arginine depletion on *Tsc1* KO HK2 cells. *Tsc1* KO and control HK2 cells were cultured in Dulbecco's modified Eagle's medium (DMEM) with or without L-arginine. The cell cycle was monitored using propidium iodide staining and FACS analysis. Quantitative analysis revealed that arginine depletion significantly reduced the proportion of cells in the G2M phase while increasing the proportion of cells in the G0/G1 phase, indicating cell-cycle arrest (Figures 5A and 5B). Furthermore, the effect of arginine depletion on cell cycle and proliferation was reinforced in *Tsc1* KO HEK293 cells, indicating that arginine depletion induces cell-cycle arrest and increases cell population at the G0/G1 phase (Figures S4B and S4C). We previously showed that *Tsc1* KO PTCs exhibit hyperactivation of the mTORC1 pathway, together with elevated c-Myc and NF-κB P65 subunit protein levels.[15] Conversely, arginine depletion in *Tsc1* KO HK2 cells and *Tsc1* KO HEK293 cells reduced mTORC1 activity, c-Myc, and NF-κB P65 subunit protein expression levels in TSC1 KO cell and to a lesser extent in control cells (Figures 5C and S5A–S5D). Taken together, these results indicate that the arginine biosynthesis pathway is a pivotal metabolic pathway essential for cell proliferation and TSC-associated cellular signaling.

Arginine deprivation ameliorates cyst formation in *Tsc1* knockout mice

To understand the effect of arginine depletion *in vivo* and its role in TSC-associated cyst development and growth, we mated *Tsc1*$^{fl/fl}$ female mice with *Six2 Cre*$^{tg/+}$ *Tsc1*$^{fl/+}$ males, as described above. After conception, as identified by a vaginal plaque, the pregnant *Tsc1*$^{fl/fl}$ females were assigned to either arginine-deficient or control diet up to the delivery date (P0), when the kidneys were excised. The groups did not differ in litter size or kidney-to-body weight ratio (Figures S6A–S6C). Histopathology analysis indicated a significant reduction in cyst overload in kidneys obtained from *Tsc1* KO mice fed an arginine-deficient diet (Figures 6A and 6B). Furthermore, the reduction in the cystic overload in *Tsc1* KO mice fed an arginine-deficient diet was associated with reduced mTORC1 activity (Figures 6C, 6D, S7A, and S7B), cell proliferation (Figures 6E and 6F) and c-Myc protein expression (Figures 7A, 7B, and S7C), as measured by immunostaining and WB analysis of these kidneys.

Moreover, the arginine-deficient diet prevented the increase in NF-κB P65 subunit protein expression in TSC cyst-lining cells (Figures 7C, 7D, and S7D), which was associated with a decline in kidney mononuclear infiltration of *Tsc1* KO kidneys, upon arginine-deficient diet treatment as was demonstrated by FACS analysis of F4/80^{+} cells in dissociated kidneys (Figures 7E and

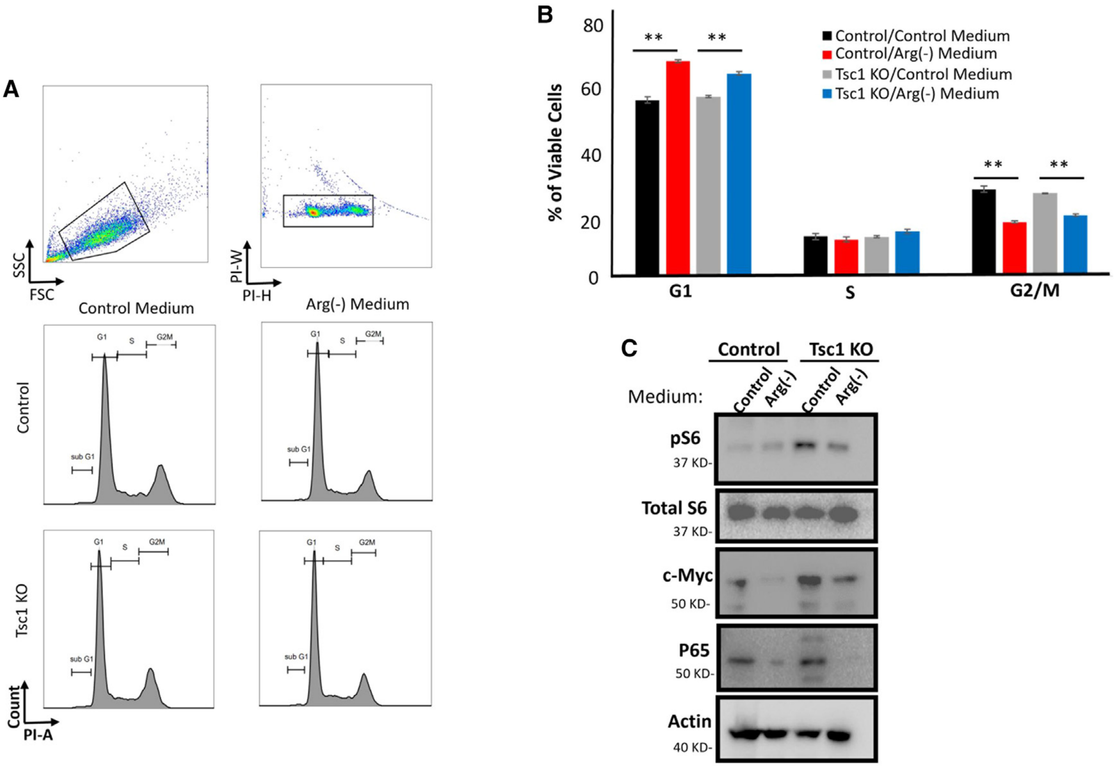

Figure 5. Arginine depletion *in vitro* induces cell-cycle arrest and attenuates TSC-associated signaling

(A) Control and *Tsc1* KO HK2 cells were incubated with either control or arginine-free medium for 10 days. Cells were harvested and fixed, and the cell cycle was monitored by propidium iodide flow cytometry-based analysis.

(B) Quantification of data in A (n = 6 biological replicates), $*p < 0.05$.

(C) Western blot analysis for ASS1, pS6, c-Myc, P65, and actin protein expression in control and *Tsc1* KO HK2, representative of 2 independent experiments with similar results.

See also Figures S4 and S5.

7F). The downregulation in protein expression of c-Myc and P65 was not observed in the corresponding mRNA levels, pointing to post-transcriptional regulation (Figure S8). Even though arginine depletion inhibited mTORC1 activation, it did not affect ASS1 expression (Figures S7D and S9).

To better understand the role of ASS1 in TSC-associated cellular signaling and cell cycle, control and *Tsc1* KO HK2 cells were infected with either control or ASS1 targeted short hairpin RNA (shRNA) containing lentivirus particles, and cultured with control high glucose DMEM media. ASS1 knockdown (KD) reduced mTORC1 activity, c-Myc, and NF-κB P65 subunit protein expression levels in control and *Tsc1* KO cells, indicating that ASS1 expression is a pivotal directly contributing to TSC-associated cellular signaling (Figure S10A). However, under the same conditions, ASS1 KD had a minimal effect on cell proliferation on both control and *Tsc1* KO HK2 cells, presumably because of the high levels of external arginine availability in the growth medium (Figures S10B and S10C).

Altogether, these results indicate that the arginine biosynthesis pathway plays a crucial role in the process of TSC-associated cystogenesis and that arginine deprivation may alleviate cyst development directly by altering PTC cell signaling, inflammation, and cell cycle.

DISCUSSION

Cystic kidney disease is a leading cause of CKD in patients with TSC disease. Here we studied the metabolic changes in TSC kidneys mouse model and especially in PTCs, and demonstrated that arginine availability has a role in the pathogenesis of TSC cystic kidney disease. We show that *Tsc1* ablation in NPCs leads to dramatic changes in a substantial number of metabolites and to perturbations in significant metabolic pathways in *Tsc1* KO kidneys. mTOR inhibition during pregnancy in *Tsc1* KO mice reversed metabolic changes, secondary to mTORC1 hyperactivation. However, some metabolites remained unresponsive to mTORC1 inhibition, indicating that, in part, these metabolic changes are mTORC1 independent. The metabolic changes were observed in whole kidney extracts and more so in sorted PTCs.

We previously showed that cysts arise from the PTCs population in the *Tsc1* KO mouse model. Other studies have previously used targeted deletions in other nephron segments, such as intercalated cells, for studying kidney disease.[9,42,43] On the basis of our metabolomic and bioinformatic analysis, we identified changes in arginine metabolism and overexpression of ASS1, a rate-limiting enzyme in the arginine biosynthetic pathway.

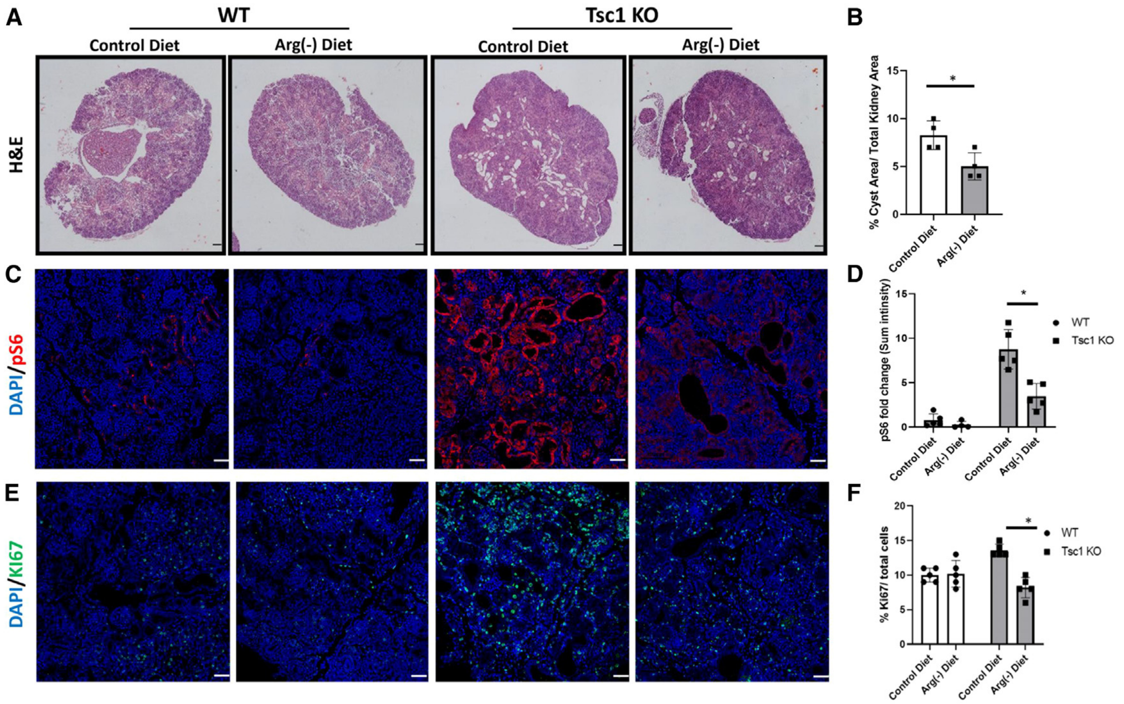

Figure 6. Arginine depletion ameliorates cyst development in *Tsc1* KO mice

(A) Representative H&E staining of kidney sections from WT (*Tsc1*$^{fl/fl}$) and *Tsc1* KO pups from *Tsc1*$^{fl/fl}$ mothers fed either control or arginine-deficient diets at P0, showing reduced cyst formation in kidneys of *Tsc1* KO pups fed an arginine-deficient diet. Scale bar: 500 μm.

(B–E) Quantification of the cyst area per section in the different groups as in (A). WT pups were fed a control diet (n = 5 biological replicates) and an arginine-deficient diet (n = 4 biological replicates). *Tsc1* KO pups fed a control diet (n = 5 biological replicates) and arginine-deficient diet (n = 5 biological replicates), *$p < 0.05$. Kidney sections, as in (A), were immunostained for pS6 (C) and for the proliferation marker Ki67 (E). Scale bar: 50 μm.

(D) Quantification of sum fluorescence intensity as in (C). *$p < 0.05$.

(F) Quantification of Ki67-positive cells compared with total cells (DAPI positive cells) as in (E). *$p < 0.05$.

See also Figures S6, S7, and S9.

These changes were observed in transcript and protein levels both *in vitro* in a human cell line of PTCs and *in vivo* in mouse and human TSC sections. The increase in ASS1 levels correlated with mTORC1 activity, as demonstrated by a decrease in ASS1 levels in a PTC cell line incubated with rapamycin. Arginine depletion in PTCs prevented the activation of signaling pathways previously shown to contribute to the cystogenic process, such as c-Myc and P65.[15] Arginine depletion also prevented the increase in mTORC1 activity and pathogenic changes in the cell cycle. Interestingly, feeding pregnant mice carrying TSC embryos with an arginine-depleted diet substantially reduced the TSC cystic load, along with a decline in mTORC1 activity and cystogenic pathways.

Arginine is a semi-essential amino acid because *de novo* arginine synthesis can be insufficient during stress, inflammation, and early embryonic development. In these cases, arginine availability depends on nutritional supply and protein degradation.[44] The synthesis of arginine occurs mainly in the kidney using the intestinal-renal axis. Citrulline, produced primarily by the small intestine, is taken up by PTCs of the kidney and is efficiently converted to arginine by the sequential action of ASS1 and ASL to meet the needs for diverse cellular functions.[45] Arginine is necessary for homeostasis as a precursor for protein synthesis, nitric oxide production, proline, creatine, agmatine, and polyamine synthesis.[40,46] Arginine is also an immune modulator through its effects on T cell activation, promoting the generation of central memory-like T cells endowed with higher survival capacity and anti-tumor activity,[38] and as an essential factor for maturation of the T cell receptor ζ-chain (TCRζ), necessary for T cells to interact with antigens.[47] In macrophages, extracellular arginine is essential for NO production in response to different pro-inflammatory stimuli and is crucial for macrophage M1/M2 polarization.[37,39] We previously showed that inflammatory reactions, mainly induced by macrophages, are involved in TSC cystogenesis.[15] Indeed, arginine depletion, both *in vitro* and *in vivo,* reduced P65 expression and macrophage infiltration in T*sc1* KO kidneys, implying reduced inflammatory response. Of note, some impact of arginine deprivation is noticed on c-Myc and P65 in the control cells, implying an additional non-TSC-mediated effect of arginine depletion. An interaction between arginine metabolism and mTORC1 activity was previously suggested.[46,48] Arginine may mediate its effects on mTOR activation by disrupting the interaction between TSC and mTORC1, activating Rag GTPase required for recruitment of mTORC1 complex to the lysosomal surface, and disrupting the CASTOR complex, regulating mTORC1 activation.[48,49] In this regard, our results support this notion by indicating that arginine depletion *in vivo* and *in vitro* tampers with mTOR activation.

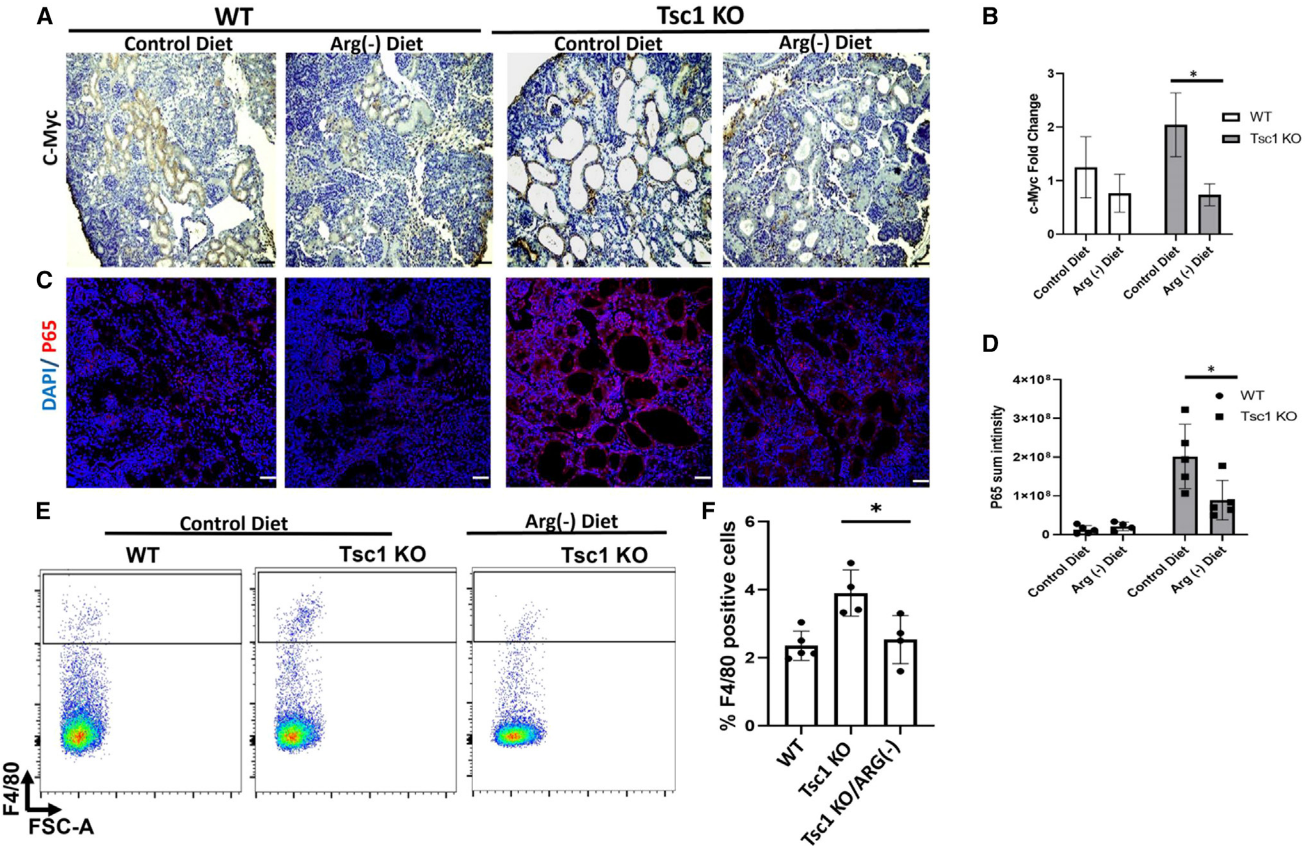

Figure 7. Arginine depletion attenuates cyst-associated c-Myc and P65 expression and reduces mononuclear cell infiltration *in vivo* in *Tsc1* KO mice

(A) Kidney sections from WT and *Tsc1* KO P0 pups from *Tsc1* $^{fl/fl}$ mothers fed either control or arginine-deficient diets were immunostained for c-Myc.

(B) Quantification of immunohistochemistry staining as in (A). $*p < 0.05$.

(C) Sections, as in (A), were immunostained for P65, showing upregulation in P65 expression in *Tsc1* KO kidneys and downregulation upon treatment with the arginine-deficient diet.

(D) Quantification of sum fluorescence intensity as in (C). WT pups fed a control diet (n = 5 biological replicates) and arginine-deficient diet (n = 4 biological replicates). *Tsc1* KO pups fed a control diet (n = 5 biological replicates) and arginine-deficient diet (n = 5 biological replicates), $*p < 0.05$.

(E) As indicated, the kidney of WT and Tsc1 KO mice subjected to either control or arginine-depleted diet were dissociated and stained with APC-conjugated F4/80 and subjected to FACS analysis. Representative FACS analysis indicating the F4/80^{+} cells in each sample.

(F) Quantification of the percentage of F4/80+ cells in each group as in (E). WT pups fed a control diet (n = 5 biological replicates), *Tsc1* KO pups fed a control diet (n = 4 biological replicates), and *Tsc1* KO pups treated with arginine-deficient diet (n = 3 biological replicates), $*p < 0.05$.

See also Figures S7–S10.

Arginine also serves as a cell signaling modulator for the activation of MAPK as well as for the activation of the transmembrane G protein-coupled receptors (GPCRs), which are responsible for signal transduction into the intracellular space.[37,39,50,51] Arginine was also implicated in activating different transcription factors by direct interaction and protein conformational change.[38] Here we show that c-Myc and P65 expression are directly governed by arginine availability and ASS1 expression, which could result from direct interaction or altered PTC cell signaling inducing c-Myc and P65 upregulation. ASS1 expression is controlled by c-Myc and HIF-1α interacting with an E-box element located at the ASS1 gene promoter.[52,53] Moreover, the ASS1 seems necessary for c-Myc expression, pointing to a direct cross-talk. Indeed, the kidneys of a TSC mouse model express high levels of ASS1 in correlation with elevated c-Myc expression. Nevertheless, in our system, arginine depletion and c-Myc downregulation were unable to downregulate ASS1 expression, which may imply a post-transcriptional mechanism regulating ASS1 expression.

Arginine is supplied in the human body mainly from the dietary resources and limited intrinsic recycling mediated by ASS1.[26] ASS1 levels are increased in various tumor types and restricting arginine metabolism by the diet or arginine catabolizing agents can be effective.[26,27,54,55] No adverse effects were reported in limiting arginine in the diet.[56] In our study, arginine depletion in mice had no visible effect on mice litter size, offspring weight, or kidney-to-body weight ratio. However, other side effects of arginine depletion during pregnancy can be detected at later stages and need further investigation.

Table 1. Oligonucleotide sequences, related to STAR Methods

Oligo name	Sequence (5′-3′)	Batch no.
Mouse ASS1 forward primer	ATGACCAGGTCCGCTTTGAG	RE00578544
Mouse ASS1 reverse primer	GGGGATTCCGTGTTGCTTTG	RE00578545
Human ASS1 forward primer	GCTTATAACCTGGGATGGGCA	RE00600859
Human ASS1 reverse primer	TTGCTGGACATAGCGTCTGG	RE00600860
Mouse GAPDH forward primer	GGGTCCCAGCTTAGGTTCAT	RE00621849
Mouse GAPDH reverse primer	CCCAATACGGCCAAATCCGT	RE00621850
Human GAPDH forward primer	GAAAGCCTGCCGGTGACTAA	RE00599424
Human GAPDH reverse primer	GCCCAATACGACCAAATCAGAG	RE00599424
Mouse Six2 Cre forward primer	GCATTACCGGTCGATGCAACGAGTGATGAG	RE00378000
Mouse Six2 Cre reverse primer	GAGTGAACGAACCTGGTCGAAATCAGTGCG	RE00378001
Mouse Tsc1 forward primer	CAGCTCCGACCATGAAGTG	RE00377994
Mouse Tsc1 reverse primer	AGGAGGCCTCTTCTGCTACC	RE00377993
Mouse P65 forward primer	CCCTGACCATGGACGATCTG	RE00732728
Mouse P65 reverse primer	TGCTTCGGCTGTTCGATGAT	RE00732729
Mouse cMyc forward primer	GTTGGAAACCCCGCAGACAG	RE00732730
Mouse cMyc reverse primer	ATAGGGCTGTACGGAGTCGT	RE00732731

Our findings indicate that arginine biosynthesis and metabolism pathways are crucial factors in mediating the TSC-associated cystogenic process. Our findings suggest that targeting arginine metabolism may alleviate TSC-associated cystogenesis by derangement of the TSC-associated cell signaling in PTCs via a reduction in mTORC1, c-Myc, and P65 signaling, lessening the inflammatory response and decreasing cystic cell proliferation. We suggest that arginine deprivation-based therapies could be relevant in treating TSC-associated kidney disease.

Limitations of the study

The mouse model used in this study is based on *Tsc1* deletion in NPCs differentiating into the majority of the cell population in the nephron. However, the study's findings cannot be extrapolated to TSC cystic kidney disease caused by *Tsc2* deletion. The study does not use experimental models with deletions in different cell populations in the kidney. Furthermore, the mouse model used in this study has an early and aggressive presentation, which does not reflect the clinical presentation of heterozygous mutation of TSC genes that characterize the human disease. Therefore, other models should be used to further confirm the study's findings.

The interpretation of the findings from human samples in this study is limited because of the low availability of TSC kidney samples, and the study includes only human embryonic tissues. To increase the validity of these findings in human TSC patients, further studies are required, such as histopathologic studies of adult TSC kidneys and experiments examining the effect of low arginine or targeted interventions on arginine metabolism in TSC patients. These studies would provide more comprehensive insights into the disease's pathophysiology and potential therapeutic interventions.

STAR★METHODS

Detailed methods are provided in the online version of this paper and include the following:

- KEY RESOURCES TABLE
- RESOURCE AVAILABILITY
  - Lead contact
  - Materials availability
  - Data and code availability
- EXPERIMENTAL MODEL AND SUBJECT DETAILS
  - Human kidney section samples
  - Animals
  - Study approval
  - Cell lines
- METHOD DETAILS
  - Section preparation, immunostaining, and analysis
  - Cystic index
  - Western Blotting
  - RNA isolation and c-DNA preparation
  - FACS-based cell sorting for kidney proximal tubule cell (PTCs) isolation
  - Cell cycle analysis
  - Metabolic analysis
  - LC-MS metabolomics analysis
  - Metabolomic statistical analysis
  - Canonical pathway enrichment analysis
- QUANTIFICATION AND STATISTICAL ANALYSIS

SUPPLEMENTAL INFORMATION

Supplemental information can be found online at https://doi.org/10.1016/j.xcrm.2023.101073.

ACKNOWLEDGMENTS

This work was supported by grants from the Israel Science Foundation, Israel (2030/21 to M.N. and 2358/18 to O.V.), the TSC Alliance Research grant , USA (O.V.), and the Research Authority of Hadassah Hebrew University Medical Center, Israel (O.V.). O.V. and M.N. are Wohl's Translation Research Institute research associates at Hadassah-Hebrew University Medical Center. The graphical abstract was created using BioRender.com. In addition, we thank Prof. Sharona Elgavish and Dr. Hadar Benyamini at the Hebrew University

bioinformatics core facility for conducting the bioinformatics analysis to interpret our data. Finally, we thank the reviewers for taking the time and effort to review the manuscript. We sincerely appreciate all your valuable comments and suggestions, which helped us improve the manuscript's quality.

AUTHOR CONTRIBUTIONS

M.N. and O.V. conceived the study, designed the experiment, and wrote the manuscript. M.N., A.A., and L.W. designed and conducted most of the experiments and supervised the rest of the experiments. H.P.C. conducted the cellular infections. I.A. and B.A. conducted metabolomics analysis experiments. E.G. helped conceive the study and critically reviewed the manuscript. I.Z.B.-D. helped conceive the study, performed the bioinformatics and statistical analysis, and critically reviewed the manuscript.

DECLARATION OF INTERESTS

The authors declare no competing interests.

Received: July 13, 2022
Revised: March 2, 2023
Accepted: May 15, 2023
Published: June 7, 2023

REFERENCES

1. Henske, E.P., Jóźwiak, S., Kingswood, J.C., Sampson, J.R., and Thiele, E.A. (2016). Tuberous sclerosis complex. Nat. Rev. Dis. Prim. *2*, 16035.
2. Salussolia, C.L., Klonowska, K., Kwiatkowski, D.J., and Sahin, M. (2019). Genetic etiologies, diagnosis, and treatment of tuberous sclerosis complex. Annu. Rev. Genom. Hum. Genet. *20*, 217–240.
3. Yang, H., Yu, Z., Chen, X., Li, J., Li, N., Cheng, J., Gao, N., Yuan, H.X., Ye, D., Guan, K.L., and Xu, Y. (2021). Structural insights into tsc complex assembly and gap activity on rheb. Nat. Commun. *12*, 339.
4. Jozwiak, J. (2006). Hamartin and tuberin: working together for tumour suppression. Int. J. Cancer *118*, 1–5.
5. Benvenuto, G., Li, S., Brown, S.J., Braverman, R., Vass, W.C., Cheadle, J.P., Halley, D.J., Sampson, J.R., Wienecke, R., and DeClue, J.E. (2000). The tuberous sclerosis-1 (tsc1) gene product hamartin suppresses cell growth and augments the expression of the tsc2 product tuberin by inhibiting its ubiquitination. Oncogene *19*, 6306–6316.
6. Giannikou, K., Malinowska, I.A., Pugh, T.J., Yan, R., Tseng, Y.Y., Oh, C., Kim, J., Tyburczy, M.E., Chekaluk, Y., Liu, Y., et al. (2016). Whole exome sequencing identifies tsc1/tsc2 biallelic loss as the primary and sufficient driver event for renal angiomyolipoma development. PLoS Genet. *12*, e1006242.
7. Amin, S., Lux, A., Calder, N., Laugharne, M., Osborne, J., and O'callaghan, F. (2017). Causes of mortality in individuals with tuberous sclerosis complex. Dev. Med. Child Neurol. *59*, 612–617.
8. Gallo-Bernal, S., Kilcoyne, A., Gee, M.S., and Paul, E. (2022). Cystic kidney disease in tuberous sclerosis complex: current knowledge and unresolved questions. Pediatr. Nephrol. https://doi.org/10.1007/s00467-022-05820-x.
9. Bissler, J.J., Zadjali, F., Bridges, D., Astrinidis, A., Barone, S., Yao, Y., Redd, J.R., Siroky, B.J., Wang, Y., Finley, J.T., et al. (2019). Tuberous sclerosis complex exhibits a new renal cystogenic mechanism. Phys. Rep. *7*, e13983.
10. Vekeman, F., Magestro, M., Karner, P., Duh, M.S., Nichols, T., van Waalwijk van Doorn-Khosrovani, S.B., and Zonnenberg, B.A. (2015). Kidney involvement in tuberous sclerosis complex: the impact on healthcare resource use and costs. J. Med. Econ. *18*, 1060–1070.
11. Kingswood, C., Bolton, P., Crawford, P., Harland, C., Johnson, S.R., Sampson, J.R., Shepherd, C., Spink, J., Demuth, D., Lucchese, L., et al. (2016). The clinical profile of tuberous sclerosis complex (tsc) in the United Kingdom: a retrospective cohort study in the clinical practice research datalink (cprd). Eur. J. Paediatr. Neurol. *20*, 296–308.
12. Kingswood, J.C., Nasuti, P., Patel, K., Myland, M., Siva, V., and Gray, E. (2016). The economic burden of tuberous sclerosis complex in UK patients with renal manifestations: a retrospective cohort study in the clinical practice research datalink (cprd). J. Med. Econ. *19*, 1116–1126.
13. Siroky, B.J., Yin, H., and Bissler, J.J. (2011). Clinical and molecular insights into tuberous sclerosis complex renal disease. Pediatr. Nephrol. *26*, 839–852.
14. Dixon, B.P., Hulbert, J.C., and Bissler, J.J. (2011). Tuberous sclerosis complex renal disease. Nephron Exp. Nephrol. *118*, e15–e20.
15. Nechama, M., Makayes, Y., Resnick, E., Meir, K., and Volovelsky, O. (2020). Rapamycin and dexamethasone during pregnancy prevent tuberous sclerosis complex-associated cystic kidney disease. JCI Insight *5*, e136857.
16. Zadjali, F., Kumar, P., Yao, Y., Johnson, D., Astrinidis, A., Vogel, P., Gross, K.W., and Bissler, J.J. (2020). Tuberous sclerosis complex axis controls renal extracellular vesicle production and protein content. Int. J. Mol. Sci. *21*, 1729.
17. Sager, R.A., Woodford, M.R., and Mollapour, M. (2018). The mtor independent function of tsc1 and fnips. Trends Biochem. Sci. *43*, 935–937.
18. Woodford, M.R., Hughes, M., Sager, R.A., Backe, S.J., Baker-Williams, A.J., Bratslavsky, M.S., Jacob, J.M., Shapiro, O., Wong, M., Bratslavsky, G., et al. (2019). Mutation of the co-chaperone tsc1 in bladder cancer diminishes hsp90 acetylation and reduces drug sensitivity and selectivity. Oncotarget *10*, 5824–5834.
19. Woodford, M.R., Backe, S.J., Sager, R.A., Bourboulia, D., Bratslavsky, G., and Mollapour, M. (2021). The role of heat shock protein-90 in the pathogenesis of birt-hogg-dubé and tuberous sclerosis complex syndromes. Urol. Oncol. *39*, 322–326.
20. Woodford, M.R., Sager, R.A., Marris, E., Dunn, D.M., Blanden, A.R., Murphy, R.L., Rensing, N., Shapiro, O., Panaretou, B., Prodromou, C., et al. (2017). Tumor suppressor tsc1 is a new hsp90 co-chaperone that facilitates folding of kinase and non-kinase clients. EMBO J. *36*, 3650–3665.
21. Lai, M., Zou, W., Han, Z., Zhou, L., Qiu, Z., Chen, J., Zhang, S., Lai, P., Li, K., Zhang, Y., et al. (2021). Tsc1 regulates tight junction independent of mtorc1. Proc. Natl. Acad. Sci. USA *118*. e2020891118.
22. Volovelsky, O., Nguyen, T., Jarmas, A.E., Combes, A.N., Wilson, S.B., Little, M.H., Witte, D.P., Brunskill, E.W., and Kopan, R. (2018). Hamartin regulates cessation of mouse nephrogenesis independently of mtor. Proc. Natl. Acad. Sci. USA *115*, 5998–6003.
23. Karbowniczek, M., Zitserman, D., Khabibullin, D., Hartman, T., Yu, J., Morrison, T., Nicolas, E., Squillace, R., Roegiers, F., and Henske, E.P. (2010). The evolutionarily conserved tsc/rheb pathway activates notch in tuberous sclerosis complex and drosophila external sensory organ development. J. Clin. Invest. *120*, 93–102.
24. Li, C., Liu, X., Liu, Y., Zhang, E., Medepalli, K., Masuda, K., Li, N., Wikenheiser-Brokamp, K.A., Osterburg, A., Borchers, M.T., et al. (2017). Tuberin regulates prostaglandin receptor-mediated viability, via rheb, in mtorc1-hyperactive cells. Mol. Cancer Res. *15*, 1318–1330.
25. Hartman, T.R., Liu, D., Zilfou, J.T., Robb, V., Morrison, T., Watnick, T., and Henske, E.P. (2009). The tuberous sclerosis proteins regulate formation of the primary cilium via a rapamycin-insensitive and polycystin 1-independent pathway. Hum. Mol. Genet. *18*, 151–163.
26. Trott, J.F., Hwang, V.J., Ishimaru, T., Chmiel, K.J., Zhou, J.X., Shim, K., Stewart, B.J., Mahjoub, M.R., Jen, K.Y., Barupal, D.K., et al. (2018). Arginine reprogramming in adpkd results in arginine-dependent cystogenesis. Am. J. Physiol. Ren. Physiol. *315*, F1855–F1868.
27. Podrini, C., Rowe, I., Pagliarini, R., Costa, A.S.H., Chiaravalli, M., Di Meo, I., Kim, H., Distefano, G., Tiranti, V., Qian, F., et al. (2018). Dissection of metabolic reprogramming in polycystic kidney disease reveals coordinated rewiring of bioenergetic pathways. Commun. Biol. *1*, 194.

28. Menezes, L.F., and Germino, G.G. (2019). The pathobiology of polycystic kidney disease from a metabolic viewpoint. Nat. Rev. Nephrol. *15*, 735–749.
29. Li, X. (2015). Polycystic Kidney Disease (Codon Publications). https://doi.org/10.15586/codon.pkd.2015.
30. Flowers, E.M., Sudderth, J., Zacharias, L., Mernaugh, G., Zent, R., DeBerardinis, R.J., and Carroll, T.J. (2018). Lkb1 deficiency confers glutamine dependency in polycystic kidney disease. Nat. Commun. *9*, 814.
31. Medvetz, D., Priolo, C., and Henske, E.P. (2015). Therapeutic targeting of cellular metabolism in cells with hyperactive mtorc1: a paradigm shift. Mol. Cancer Res. *13*, 3–8.
32. Hilliard, S., Tortelote, G., Liu, H., Chen, C.H., and El-Dahr, S.S. (2022). Single-cell chromatin and gene-regulatory dynamics of mouse nephron progenitors. J. Am. Soc. Nephrol. *33*, 1308–1322.
33. Morris, C.R., Hamilton-Reeves, J., Martindale, R.G., Sarav, M., and Ochoa Gautier, J.B. (2017). Acquired amino acid deficiencies: a focus on arginine and glutamine. Nutr. Clin. Pract. *32*, 30S–47S.
34. Oh, H.S., Oh, S.K., Lee, J.S., Wu, C., and Lee, S.J. (2017). Effects of l-arginine on growth hormone and insulin-like growth factor 1. Food Sci. Biotechnol. *26*, 1749–1754.
35. Liang, M., Wang, Z., Li, H., Liu, B., and Yang, L. (2022). L-arginine prevents 4-hydroxy-2-nonenal accumulation and depresses inflammation via inhibiting nf-κb activation. J. Biochem. Mol. Toxicol. *36*, e23087.
36. Ge, Y., Li, F., He, Y., Cao, Y., Guo, W., Hu, G., Liu, J., and Fu, S. (2022). L-arginine stimulates the proliferation of mouse mammary epithelial cells and the development of mammary gland in pubertal mice by activating the gprc6a/pi3k/akt/mtor signalling pathway. J. Anim. Physiol. Anim. Nutr. *106*, 1383–1395.
37. Pekarova, M., and Lojek, A. (2015). The crucial role of l-arginine in macrophage activation: what you need to know about it. Life Sci. *137*, 44–48.
38. Geiger, R., Rieckmann, J.C., Wolf, T., Basso, C., Feng, Y., Fuhrer, T., Kogadeeva, M., Picotti, P., Meissner, F., Mann, M., et al. (2016). L-arginine modulates t cell metabolism and enhances survival and anti-tumor activity. Cell *167*, 829–842.e13.
39. Buchmüller-Rouiller, Y., Corradin, S.B., and Mauël, J. (1992). Macrophage activation for intracellular killing as induced by a ca2+ ionophore. Dependence on l-arginine-derived nitrogen oxidation products. Biochem. J. *284*, 387–392.
40. Szefel, J., Danielak, A., and Kruszewski, W.J. (2019). Metabolic pathways of l-arginine and therapeutic consequences in tumors. Adv. Med. Sci. *64*, 104–110.
41. Hernandez, J.O.R., Wang, X., Vazquez-Segoviano, M., Lopez-Marfil, M., Sobral-Reyes, M.F., Moran-Horowich, A., Sundberg, M., Lopez-Cantu, D.O., Probst, C.K., Ruiz-Esparza, G.U., et al. (2021). A tissue-bioengineering strategy for modeling rare human kidney diseases in vivo. Nat. Commun. *12*, 6496.
42. Iwata, W., Unoki-Kubota, H., Kato, H., Shimizu, A., Matsumoto, M., Imasawa, T., Igarashi, A., Matsumoto, K., Noda, T., Terauchi, Y., et al. (2020). Podocyte-specific deletion of tubular sclerosis complex 2 promotes focal segmental glomerulosclerosis and progressive renal failure. PLoS One *15*, e0229397.
43. Ren, S., Luo, Y., Chen, H., Warburton, D., Lam, H.C., Wang, L.L., Chen, P., Henske, E.P., and Shi, W. (2016). Inactivation of tsc2 in mesoderm-derived cells causes polycystic kidney lesions and impairs lung alveolarization. Am. J. Pathol. *186*, 3261–3272.
44. Luiking, Y.C., Ten Have, G.A.M., Wolfe, R.R., and Deutz, N.E.P. (2012). Arginine de novo and nitric oxide production in disease states. Am. J. Physiol. Endocrinol. Metab. *303*, E1177–E1189.
45. Haines, R.J., Pendleton, L.C., and Eichler, D.C. (2011). Argininosuccinate synthase: at the center of arginine metabolism. Int. J. Biochem. Mol. Biol. *2*, 8–23.
46. Morris, S.M. (2016). Arginine metabolism revisited. J. Nutr. *146*, 2579S–2586S.
47. Taheri, F., Ochoa, J.B., Faghiri, Z., Culotta, K., Park, H.J., Lan, M.S., Zea, A.H., and Ochoa, A.C. (2001). L-arginine regulates the expression of the t-cell receptor zeta chain (cd3zeta) in jurkat cells. Clin. Cancer Res. *7*, 958s–965s.
48. Shimobayashi, M., and Hall, M.N. (2016). Multiple amino acid sensing inputs to mtorc1. Cell Res. *26*, 7–20.
49. Takahara, T., Amemiya, Y., Sugiyama, R., Maki, M., and Shibata, H. (2020). Amino acid-dependent control of mtorc1 signaling: a variety of regulatory modes. J. Biomed. Sci. *27*, 87.
50. Morris, S.M. (2010). Arginine: master and commander in innate immune responses. Sci. Signal. *3*, pe27.
51. Mieulet, V., Yan, L., Choisy, C., Sully, K., Procter, J., Kouroumalis, A., Krywawych, S., Pende, M., Ley, S.C., Moinard, C., and Lamb, R.F. (2010). Tpl-2-mediated activation of mapk downstream of tlr4 signaling is coupled to arginine availability. Sci. Signal. *3*, ra61.
52. Long, Y., Tsai, W.B., Chang, J.T., Estecio, M., Wangpaichitr, M., Savaraj, N., Feun, L.G., Chen, H.H.W., and Kuo, M.T. (2016). Cisplatin-induced synthetic lethality to arginine-starvation therapy by transcriptional suppression of ass1 is regulated by dec1, hif-1α, and c-myc transcription network and is independent of ass1 promoter dna methylation. Oncotarget *7*, 82658–82670.
53. Brashears, C.B., Barlin, M., Ehrhardt, W.R., Rathore, R., Schultze, M., Tzeng, S.C., Van Tine, B.A., and Held, J.M. (2020). Systems level profiling of arginine starvation reveals myc and erk adaptive metabolic reprogramming. Cell Death Dis. *11*, 662.
54. Al-Koussa, H., El Mais, N., Maalouf, H., Abi-Habib, R., and El-Sibai, M. (2020). Arginine deprivation: a potential therapeutic for cancer cell metastasis? A review. Cancer Cell Int. *20*, 150.
55. Zou, S., Wang, X., Liu, P., Ke, C., and Xu, S. (2019). Arginine metabolism and deprivation in cancer therapy. Biomed. Pharmacother. *118*, 109210.
56. Kaiser-Kupfer, M.I., Caruso, R.C., Valle, D., and Reed, G.F. (2004). Use of an arginine-restricted diet to slow progression of visual loss in patients with gyrate atrophy. Arch. Ophthalmol. *122*, 982–984.
57. Sud, M., Fahy, E., Cotter, D., Azam, K., Vadivelu, I., Burant, C., Edison, A., Fiehn, O., Higashi, R., Nair, K.S., et al. (2016). Metabolomics workbench: an international repository for metabolomics data and metadata, metabolite standards, protocols, tutorials and training, and analysis tools. Nucleic Acids Res. *44*, D463–D470.
58. Mackay, G.M., Zheng, L., van den Broek, N.J.F., and Gottlieb, E. (2015). Analysis of cell metabolism using lc-ms and isotope tracers. Methods Enzymol. *561*, 171–196.
59. Pietzke, M., and Vazquez, A. (2020). Metabolite autoplotter - an application to process and visualise metabolite data in the web browser. Cancer Metabol. *8*, 15.
60. Chong, J., Wishart, D.S., and Xia, J. (2019). Using metaboanalyst 4.0 for comprehensive and integrative metabolomics data analysis. Curr. Protoc. Bioinformatics *68*, e86.

CellPress
OPEN ACCESS

STAR★METHODS

KEY RESOURCES TABLE

REAGENT or RESOURCE	SOURCE	IDENTIFIER
Antibodies		
Rabbit anti-pS6 ribosomal protein	Cell Signaling	Cat# 2211; RRID: AB_331679
Mouse S6 Ribosomal Protein(54D2) antibody	Cell Signaling	Cat# 2317; RRID: AB_2238583
HRP-*anti*-beta-actin antibody	Abcam	Cat# ab20272; RRID: AB_445482
Rabbit anti-*c*-Myc	Cell Signaling	Cat# 5605; RRID: AB_1903938
Rabbit anti-P65	Cell Signaling	Cat# 8242; RRID: AB_10859369
Rabbit anti-ASS1	Abcam	Cat# ab191165
Rabbit anti-Tsc1	Abcam	Cat# ab227594
Mouse anti-GAPDH	EMD Millipore	Cat# MAB374; RRID: AB_2107445
PE-conjugated anti-CD133/prominin-1 antibody	Thermo Fisher Scientific	Cat# 12-1331-82; RRID: AB_465849
Rabbit anti Phosphor- 4E-BP1 (Ser65) Antibody	Cell Signaling	Cat# 9451; RRID: AB_330947
Biotinylated Lotus Tetragonolobus Lectin (LTL) antibody	Vector laboratories	Cat# B-1325; RRID: AB_2336558
Alexa Flour 594-conjucated streptavidin	Jackson ImmunoResearch	Cat# 016-580-084; RRID:AB_2337250
APC-conjugated anti-F4/80 antibody	Macs Miltenyi Biotec	Cat# 130-116-525; RRID: AB_2733417
Cy3-conjugated goat anti-rabbit antibody	Jackson ImmunoResearch	Cat# 711-165-152; RRID: AB_2307443
Cy5-conjugated goat anti-mouse antibody	Jackson ImmunoResearch	Cat# A90-516C5; AB_10630988
Histofine Simple Stain MAX PO Anti-Mouse Anti-Rabbit Antibody	N-Histofine	Cat# 414152F
Bacterial and virus strains		
CRISPR/Cas9 TSC1 gRNA	Sigma Aldrich	Clone# HSPD0000043237
pSpCas9(BB)-2A-Puro	Gift from Prof. Iddo Ben Dov, Nephrology Department, Hadassah Medical Center, Jerusalem, Israel	
shRNA ASS1	Sigma Aldrich	Clone # TRCN0000045554, TRCN0000440576
pLKO.1 *neo*	Gift from Prof.Kun Ping Lu, Western University, London, Canada	
Biological samples		
Embryonic kidney sections from human TSC and non-TSC patients	Histopathology core facility at Hadassah Medical Center, Jerusalem, Israel	
Chemicals, peptides, and recombinant proteins		
Trizol (TRI) reagent	Bio-Lab	Cat# 009010233100
HBSS	Sigma-Aldrich	Cat# H6648-500ML
DMEM modified medium	Biological Industries	Cat# 01-050-1A
Fetal bovine serum	Sigma-Aldrich	Cat# F7524-500ML
DAB reagent	Thermo-Scientific	Cat# TA-125- QHDX
Arginine-Free Modified DMEM medium	Biological Industries	Cat#06-1050-44-1-A
Protease/phosphatase inhibitors	MERCK	Cat# 4906837001
Collagenase/Dispase	Sigma Aldrich	Cat# 10269638001
Puromycin	Sigma Aldrich	Cat# P7255
Neomycin Sulfate (10 mg/mL solution)	Sigma Aldrich	Cat# N1142
Penicillin-streptomycin	Sigma Aldrich	Cat# P4333-100ML
Critical commercial assays		
High-capacity cDNA Reverse Transcription Kit containing RNase Inhibitor	Applied Biosystems	Cat# 4374966
Propidium Iodide Flow Cytometry Kit	Abcam	Cat# ab139418

(*Continued on next page*)

Continued

REAGENT or RESOURCE	SOURCE	IDENTIFIER
Deposited data		
Raw and summary metabolomics data	https://www.metabolomicsworkbench.org	https://doi.org/10.21228/M8TD8H
Experimental models: Cell lines		
Human: Proximal tubular epithelial cell line HK-2	Gift from Prof. Iddo Ben Dov, Nephrology Department, Hadassah Medical Center, Jerusalem, Israel	
Human: Embryonic kidney cell line HEK-293	Gift from Prof. Tally Naveh-Many Ph.D, Nephrology Department, Hadassah Medical Center, Jerusalem, Israel	
Experimental models: Organisms/strains		
Mouse: CD1 Tg(Six2-EGFP/cre)1Amc	Gift from Raphael Kopan's lab, Developmental biology department, Cincinnati Children's Hospital Medical Center, Ohio, USA	
Mouse*: CD1 Tsc1*$^{fl/fl}$	Gift from Raphael Kopan's lab, Developmental biology department, Cincinnati Children's Hospital Medical Center, Ohio, USA	
Oligonucleotides		
Primers for Mouse ASS1 see Table 1	Sigma-Aldrich	Cat# RE0057854-4/5
Primers for Human ASS1 see Table 1	Sigma-Aldrich	Cat# RE00600-859/860
Primers for Mouse GAPDH see Table 1	Sigma-Aldrich	Cat# RE006218-49/50
Primers for Human GAPDH see Table 1	Sigma-Aldrich	Cat# RE0059942-4/5
Primers for Mouse Six2 Cre see Table 1	Sigma-Aldrich	Cat#RE0037800-0/1
Primers for Mouse *Tsc1* see Table 1	Sigma-Aldrich	Cat# RE0037799-4/3
Primers for Mouse P65 see Table 1	Sigma-Aldrich	Cat# RE0073272-8/9
Primers for Mouse c-Myc see Table 1	Sigma-Aldrich	Cat# RE0073273-0/1
Software and algorithms		
LSRII flow cytometry and FlowJo software	The core research facility, The Hebrew University of Jerusalem, Jerusalem, Israel	https://crf.huji.ac.il/bd-lsr-ii
Ingenuity Pathway Analysis (IPA®)	QIAGEN Inc.	https://digitalinsights.qiagen.com/products-overview/discovery-insights-portfolio/content-exploration-and-databases/qiagen-ipa/
MetaboAnalyst server, version 4.0	Chong, Wishart, and Xia[60]	https://www.metaboanalyst.ca
GraphPad Prism version 8.3.0	Pediatric Nephrology Research lab, Hadassah Medical School, Jerusalem, Israel.	https://www.graphpad.com/updates/prism-830-release-notes
Thermo Xcalibur 4.4		
Thermo TraceFinder™ 4.1 software		
Metabolite-Auto Plotter 2.3	Pietzke and Vazquez[59]	
R project for statistical computing		
IMARIS 9.8.0 software	The core research facility, The Hebrew University of Jerusalem, Jerusalem, Israel	https://crf.huji.ac.il/nikon-confocal-a1r
NIS-Elements AR analysis software	The core research facility, The Hebrew University of Jerusalem, Jerusalem, Israel	https://crf.huji.ac.il/nikon-confocal-a1r
Other		
Amino Acid diet	ENVIGO	Cat# TD.01084
Arginine deficient diet	ENVIGO	Cat# TD.09152

CellPress
OPEN ACCESS

RESOURCE AVAILABILITY

Lead contact

Further information and requests for resources and reagents should be directed to and will be fulfilled by the lead contact, Dr. Oded Volovelsky (odedvo@hadassah.org.il).

Materials availability

This study did not generate new unique reagents or code.

Data and code availability

- Raw and summary metabolomics data from this study is available at the NIH Common Fund's National Metabolomics Data Repository (NMDR) Website, the Metabolomics Workbench,[57] https://www.metabolomicsworkbench.org, where it has been assigned Study IDs ST002457 and ST002458. The data can be accessed directly via its Project https://doi.org/10.21228/M8TD8H.
- This paper does not report an original code. However, an excerpt of the MetaboAnalyst R code and a respective concentration file needed to run the code can be downloaded from https://t.ly/0dGs.
- Any additional information required to reanalyze the data reported in this paper is available from the lead contact upon request

EXPERIMENTAL MODEL AND SUBJECT DETAILS

Human kidney section samples

Embryonic kidney sections from human TSC and non-TSC patients were obtained from the histopathology core facility at Hadassah Medical Center as was approved by the Hebrew University IRB committee (HMO-0516-17). All samples were de-identified and numbered before immunostaining and analysis. Two non-TSC kidney sections were obtained: 1) Week 14 of gestation (Number 237). An induced termination for suspected Cytomegaloviral infection, without specific pathologic findings in the fetus or the placenta; and 2) Perinatal death at 29 weeks of gestation due to severe Pierre Robin sequence, but without visceral congenital anomalies (Number 201).

In addition, two age-matched TSC kidney sections were obtained: 1) 12 weeks of gestation (Number 21533). An induced termination for TSC1 mutation was examined without specific pathologic findings in the material. 2) 41 weeks of gestation (Number 32). An induced termination for TSC phenotype includes multiple tubers, subependymal nodules in the brain, and multiple cardiac rhabdomyomas. The kidneys were not severely affected, with only occasional cortical cysts.

Animals

All mice were maintained in the Hebrew University Specific-Pathogen-Free (SPF) Animal Facility Unit. The following CD1 transgenic mice lines were used: Tg (Six2-EGFP/cre)1Amc (herein Six2 Cre $^{tg/+}$) and *Tsc1*$^{fl/fl}$.[4] For deletion of *Tsc1* in NPCs, 6- to 8-week-old Six2 Cre$^{tg/+}$ male mice were mated with 6- to 8-week-old *Tsc1*$^{fl/fl}$ females. 25% of the pups were expected to carry the homozygous *Tsc1* deletion (Six2 Cre$^{tg/+}$ *Tsc1*$^{fl/fl}$). The mice genotype was identified using the following primers— Six2 Cre forward: GCATTACCG GTCGATGCAACGAGTGATGAG; Six2 Cre reverse: GAGTGAACGAACCTGGTCGAAATCAGTGCG; *Tsc1* forward: CAGCTCCGACC ATGAAGTG; and *Tsc1* Reverse: AGGAGGCCTCTTCTGCTAC. The pregnancy date was determined by vaginal plug expulsion. The morning of plug detection was designated as day 0.5 of pregnancy. In our experiments, 16 pregnant *Tsc1*$^{fl/fl}$ females were separated into individual cages. Pregnant females were randomly placed either on a control diet (here in the control diet, n = 8 females) (ENVIGO, Cat #TD.01084) or an arginine deficient diet (here in Arg (–) Diet, n = 8 females) (ENVIGO, Cat #TD.09152) up to delivery date. Upon delivery (P0), newborn pups were dissected, and kidneys were excised. Newborn pups' kidneys and body weights were recorded, one kidney was fixed in fresh 4% formaldehyde in PBS for histological sectioning, and the other kidney was used for protein and RNA extraction.

Study approval

The animal study was approved by the Hebrew University Authority For Biological and Biomedical Models (Authorization number: MD-22-16835-3). The Hebrew University IRB committee approved the usage of human kidney sections in this study (HMO-0516-17).

Cell lines

The human proximal tubular epithelial cell line HK-2 and human embryonic kidney cell line HEK-293 were cultured in DMEM-modified medium (Biological Industries, Cat #01-050-1A), supplemented with 10% fetal bovine serum (Sigma Aldrich, Cat #F7524-500ML), 100 U/mL penicillin, and 100 μg/mL streptomycin (Sigma Aldrich, Cat #P4333-100ML) at 37°C in a humid atmosphere with 5% CO2. For TSC1 deletion, each of HK-2 and HEK-293 cells were infected with CRISPR/Cas9 lentiviral transduction particles containing the guide sequence: TTCCACCTCCGACGAGAGT, specifically targeting the human TSC1 gene (Sigma Aldrich,

Clone # HSPD000043237), or the control CRISPR/Cas9 lentiviral transduction particles. 48 h after infection, the cells were treated with 1ug/ml puromycin (Sigma Aldrich, Cat #P7255) for additional 7 days before GFP^{+} cells were isolated using the BD Aria III flow cytometry-based cell sorting (The core research facility, The Hebrew University of Jerusalem, Jerusalem, Israel, https://crf.huji.ac.il/bd-aria-iii). Control and *TSC1* knockout HK-2 and HEK-293 cells were then incubated for 10 days with either control Modified DMEM medium (Biological Industries, Cat #01-050-1A) or arginine-free Modified DMEM medium (Biological Industries Cat #06-1050-44-1-A). Both media were deprived of the following amino acids: alanine, asparagine, glutamic acid, glutamine, aspartic acid and proline. In some cases, as indicated, control and *Tsc1* KO HK2 cells were infected with either control or ASS1 targeting shRNA-containing particles (Sigma Aldrich, Clone # TRCN0000045554, TRCN0000440576), and selected with Neomycin Sulfate (50 μg/ml) (Sigma Aldrich, Cat #N1142) for 10 days shRNA sequences are as fallow: Clone # TRCN0000045554: GCCTGAATTCTACAACCG GTT, Clone # TRCN0000440576: CCCAAGTACAGGCGCTAATTG.

METHOD DETAILS

Section preparation, immunostaining, and analysis

Kidneys at P0 were fixed in fresh 4% formaldehyde in PBS. Kidneys were embedded in paraffin and sectioned. For general histology, tissue sections were stained with hematoxylin and eosin. Immunofluorescence/Immunohistochemistry staining was performed as previously described.[15] Briefly, paraffin-embedded tissue sections (4–6 μm) were deparaffinized, hydrated, and incubated overnight at 4C with the following antibodies, according to the manufacturer's instructions: rabbit anti-phospho S6 ribosomal protein (Cell Signaling, Cat #2211), mouse anti-Ki67 (Novus Bio, Cat #NBP2-22112), rabbit anti-*c*-Myc (cell signaling, Cat#5605), rabbit anti-P65 (cell signaling, Cat #8242) and rabbit anti-ASS1 (Abcam, Cat #ab191165). For IF staining, the sections were incubated with Cy3-conjugated goat anti-rabbit (Jackson ImmunoResearch, Cat #711-165-152) or Cy5-conjugated goat anti-mouse (Jackson ImmunoResearch, Cat #A90-516C5) antibody according to the manufacturer's instructions. For IHC, DAB reagent (Thermo-Scientific, Cat # TA-125-QHDX) was applied after incubation with Histofine Simple Stain MAX PO Anti-Mouse Anti-Rabbit Antibody (N-Histofine, Cat #414152F). Human embryonic kidney sections were stained for rabbit anti-ASS1 (Abcam, Cat #ab191165) and Anti Biotinylated Lotus Tetragonolobus Lectin (LTL) antibody (Vector Laboratories, Cat #B-1325). The sections were then incubated with either Cy3-conjugated goat anti-rabbit (Jackson ImmunoResearch, Cat #711-165-152) or Alexa Flour 594-conjugated streptavidin (Jackson ImmunoResearch, Cat #016-580-084) respectively. All sections and slides were visualized with a confocal A1R microscope and analyzed using IMARIS 9.8.0 software and NIS-Elements AR analysis software (The core research facility, The Hebrew University of Jerusalem, Jerusalem, Israel, https://crf.huji.ac.il/nikon-confocal-a1r).

Cystic index

Hematoxylin-stained sections were visualized by a Nikon TL light microscope (The core research facility, The Hebrew University of Jerusalem, Jerusalem, Israel, https://crf.huji.ac.il/nikon-fluorescent). The cyst number for a given section area and the cyst area ratio compared with the total kidney section area were evaluated using NIS-Elements AR analysis software (The core research facility, The Hebrew University of Jerusalem, Jerusalem, Israel, https://crf.huji.ac.il/nikon-confocal-a1r).

Western Blotting

Whole kidneys or HK-2 and HEK-293 cells were homogenized in ice-cold RIPA buffer containing 150 mM NaCl, 1% NP40, 0.5% sodium deoxycholate, 0.1% SDS, and 25 mM Tris (pH 7.4) supplemented with protease/phosphatase inhibitors (MERCK, Cat #4906837001). An equal amount of protein extract was analyzed by SDS-PAGE, as previously described[15] using the following antibodies according to the manufacturer's instructions: rabbit anti-pS6 ribosomal protein (Cell Signaling, Cat #2211), HRP-*anti*-beta actin antibody (Abcam, Cat #ab20272), rabbit anti-*c*-Myc, (cell signaling, Cat #5605), rabbit anti-P65 (cell signaling, Cat #8242), rabbit anti-ASS1 (Abcam, Cat #ab191165), rabbit anti-TSC1 (Abcam, Cat #ab227594), mouse anti-GAPDH (EMD Millipore, Cat #MAB374), mouse S6 Ribosomal Protein(54D2) antibody (Cell Signaling, Cat #2317) and Rabbit phosphor-4E-BP1 (Ser65) antibody (Cell Signaling, Cat #9451).

RNA isolation and c-DNA preparation

Total RNA was extracted from 50 to 100 mg of kidney samples or HK-2 cells using Trizol (TRI) reagent (Bio-Lab, Cat #009010233100) as was previously described.[15] Complementary DNA (cDNA) was synthesized from 1 μg of total RNA using a High-Capacity cDNA Reverse Transcription Kit containing RNase Inhibitor (Applied Biosystems, Cat #4374966) following the manufacturer's instructions. All primers used for qRT-PCR are summarized in Table 1.

FACS-based cell sorting for kidney proximal tubule cell (PTCs) isolation

PTCs were isolated as previously described.[15] Briefly, kidneys were excised in ice-cold HBSS buffer (Sigma-Aldrich, Cat #H6648-500ML). The kidneys were sliced and chopped into pieces (~0.5–1 mm) on ice using a surgical scalpel. The chopped kidneys were transferred into an HBSS solution containing 1 μg/μL collagenase/dispase (Sigma Aldrich, Cat #10269638001) and incubated for 25 min at 37°C. The cells were filtered through a 40-μm nylon cell strainer (Corning, Cat #431750) and washed twice with cold

HBSS. For PTC isolation, the cells were stained with PE-conjugated anti-CD133/prominin-1 antibody (Invitrogen, Cat #12-1331-82) according to the manufacturer's instructions. PE + cells were isolated by BD Aria III flow cytometry-based cell sorting (The core research facility, The Hebrew University of Jerusalem, Jerusalem, Israel, https://crf.huji.ac.il/bd-aria-iii). For FACS analysis of F4/80+ cells, kidneys were chopped as described above and stained with APC-conjugated anti-F4/80 antibody (Macs Miltenyi Biotec, Cat #130-116-525). The cells were washed twice with HBSS before analysis by LSRII flow cytometry (The core research facility, The Hebrew University of Jerusalem, Jerusalem, Israel, https://crf.huji.ac.il/bd-lsr-ii).

Cell cycle analysis

Control, *TSC1* KO HK-2, and HEK-293 cells were cultured using the previously mentioned medium as above. The cells were harvested, fixed, and stained with Propodeum iodide (Abcam, Cat #ab139418) according to manufacturer instructions. The cells were analyzed by LSRII flow cytometry and FlowJo software (The core research facility, The Hebrew University of Jerusalem, Jerusalem, Israel, https://crf.huji.ac.il/bd-lsr-ii).

Metabolic analysis

For Kidneys metabolomics profiling: snap-frozen kidneys were transferred into 0.5 mL homogenization tubes prefilled with 1.4mm ceramic beads (CK14, #P000933-LYSK0-A, Bertin corp) and cold (−20°C) metabolites extraction solvent (Methanol: acetonitrile: water at a ratio of 5:3:2 respectively). A mixture of six labeled internal standards was added to the extraction solution for quality control (13C_6-Glucose, 13C_5-Glutamine, 13C_5-Glutamate, 13C_1-Alanine, 13C_3-Pyruvate, and 13C_3-Lactate). The exact volume of each tube was adjusted according to tissue weight (average volume of 200 μL). The samples were homogenized using Precellys 24 tissue homogenizer (Bertin Technologies) precooled to 4°C (3 cycles × 30 s at 6000 rpm, with a 30 s gap between each cycle) later centrifuged at 18,000 g for 15 min at 4°C. The supernatants were transferred into HPLC glass vials and kept at −80°C before LC-MS analysis.

For cellular metabolomics profiling: Approximately 500,000 isolated PTCs in each biological sample were extracted using 200 μL of the same metabolite extract solution described above. Samples were vortexed for 15 min and centrifuged at 20,000g for 20 min at 4°C. The supernatants were transferred into HPLC glass vials and kept at −80°C before LC-MS analysis.

LC-MS metabolomics analysis

LC-MS analysis was conducted as described.[58] Briefly, Dionex Ultimate ultra-high-performance liquid chromatography (UPLC) system coupled to Orbitrap Q-Exactive mass spectrometer (Thermo Fisher Scientific) was used. The resolution was set to 35,000 at a 200 mass/charge ratio (*m/z*) with electrospray ionization and polarity switching mode to enable both positive and negative ions across a mass range of 67–1000 *m/z*. UPLC setup consisted of a ZIC-pHILIC column (SeQuant; 150 mm × 2.1 mm, 5 μm; Merck). 5 μL of cells or kidney extracts were injected using an autosampler. Compounds were separated using a 15-min gradient, starting at 20% aqueous (20 mM ammonium carbonate adjusted to pH 9.2 with 0.1% of 25% ammonium hydroxide) and 80% organic (acetonitrile), terminated with 20% acetonitrile. Flow rate and column temperature were kept at 0.2 mL/min and 45°C, respectively, for a total run time of 27 min. All metabolites were detected using mass accuracy below 5 ppm. Thermo Xcalibur 4.4 was used for data acquisition. The peak areas of different metabolites were determined using Thermo TraceFinder 4.1 software. Metabolites were identified using the exact mass of the singly charged ion and the retention time of a matching standard using an in-house library acquired by running commercial standards for all detected metabolites. The peak areas of each identified metabolite were normalized to mg tissue or cell number. Metabolite-Auto Plotter 2.3[59] was used for data visualization during data processing. Raw and summary metabolomics data from this study are available at the NIH Common Fund's National Metabolomics Data Repository (NMDR) Website, the Metabolomics Workbench,[57] https://www.metabolomicsworkbench.org where it has been assigned Study IDs ST002457 and ST002458. The data can be accessed directly via its Project https://doi.org/10.21228/M8TD8H.

Metabolomic statistical analysis

Metabolomic profiling results were further analyzed using the MetaboAnalyst web server version 4.0[60] (https://www.metaboanalyst.ca), and the respective R package (MetaboAnalystR: https://github.com/xia-lab/MetaboAnalystR), applying the following pre-analytical options: filtering – none, normalization – quantile, transformation – logarithmic, scaling – none. We used MetaboAnalyst's intrinsic single factor and multivariate statistical analyses, including fold change, unpaired t-test (applying Benjamini-Hochberg correction for multiple testing), principal component analysis (PCA), clustering dendrogram and heatmap, and the pathway enrichment analysis module. An excerpt of the R code and a respective concentration file needed to run the code can be downloaded from https://t.ly/0dGs. Additional downstream computations were conducted on R and plotted using the 'ggplot2' (scatter correlation plots) and 'ggVennDiagram' (Venn diagrams) packages. Metabolite concentration tables and results of differential concentration (expression) analyses are provided as Tables S1–S6.

Canonical pathway enrichment analysis

Canonical pathway enrichment analysis of the significantly differentially expressed genes and metabolites was carried out using the Ingenuity Pathway Analysis (IPA) (QIAGEN Inc., https://digitalinsights.qiagen.com/products-overview/discovery-insights-portfolio/content-exploration-and-databases/qiagen-ipa/).

QUANTIFICATION AND STATISTICAL ANALYSIS

The numbers of biological samples were determined based on effect size or sample variation. No statistical method was used to pre-determine the sample size. No animals or samples were excluded from any analysis. Animals were randomly assigned to groups for *in vivo* studies; no formal randomization method was applied when assigning animals for treatment. Values are reported as means ± SEM unless otherwise stated. The data were analyzed by a student's 2-tailed t-test. The significance was set at a p value of less than 0.05. The data are presented using the GraphPad Prism version 8.3.0 and the R project for statistical computing.

Cell Reports Medicine, Volume *4*

Supplemental information

Arginine depletion attenuates renal cystogenesis in tuberous sclerosis complex model

Athar Amleh, Hadass Pri Chen, Lana Watad, Ifat Abramovich, Bella Agranovich, Eyal Gottlieb, Iddo Z. Ben-Dov, Morris Nechama, and Oded Volovelsky

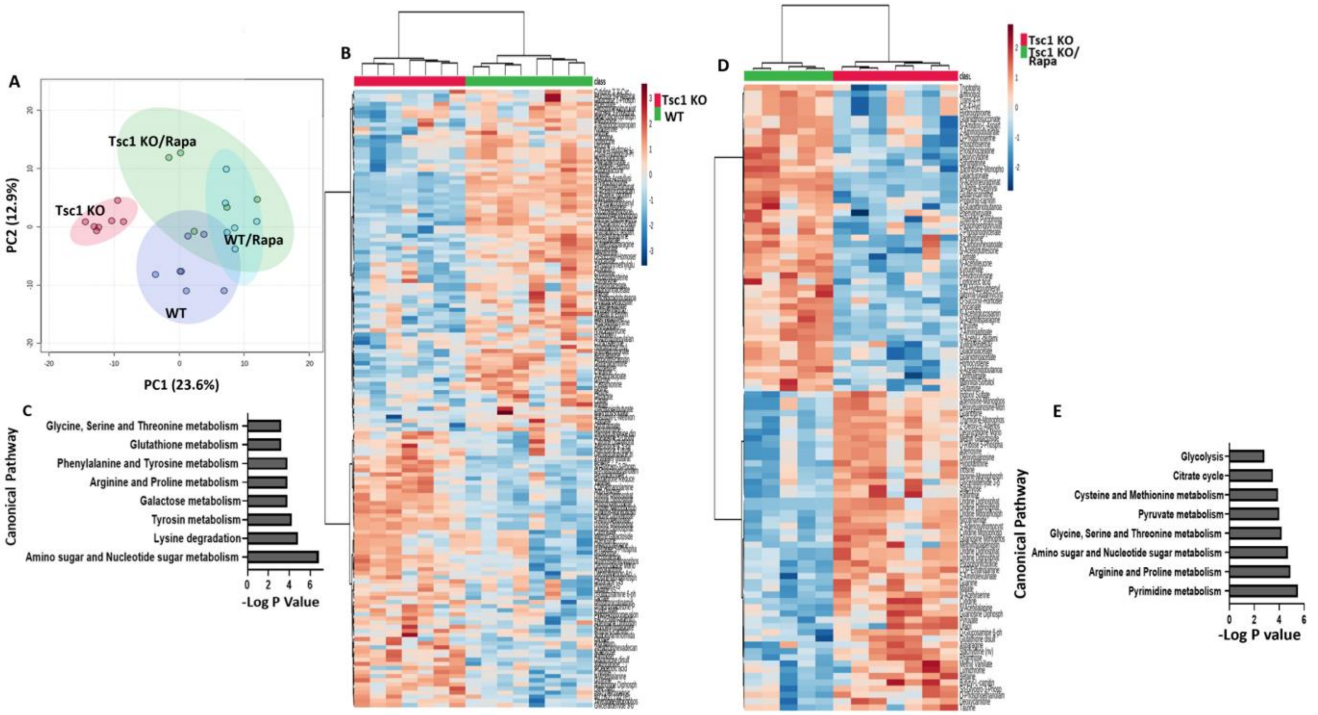

Figure S1: Rapamycin treatment alters kidney metabolic programming in *Tsc1* KO mice, Related to Figure 1.

(A) Principal components analysis plot of WT mice and *Tsc1* KO mice treated with either vehicle or rapamycin during pregnancy. Metabolic analysis of vehicle-treated *Tsc1* KO and WT mice (**B-C**) as well as *Tsc1* KO mice treated with either rapamycin or vehicle (**D-E**). (**B**) and (**D**) show heat maps constructed via hierarchical clustering based on significantly dysregulated metabolites. (**C**) and (**E**) show pathways enriched with the respective dysregulated metabolites in each comparison.

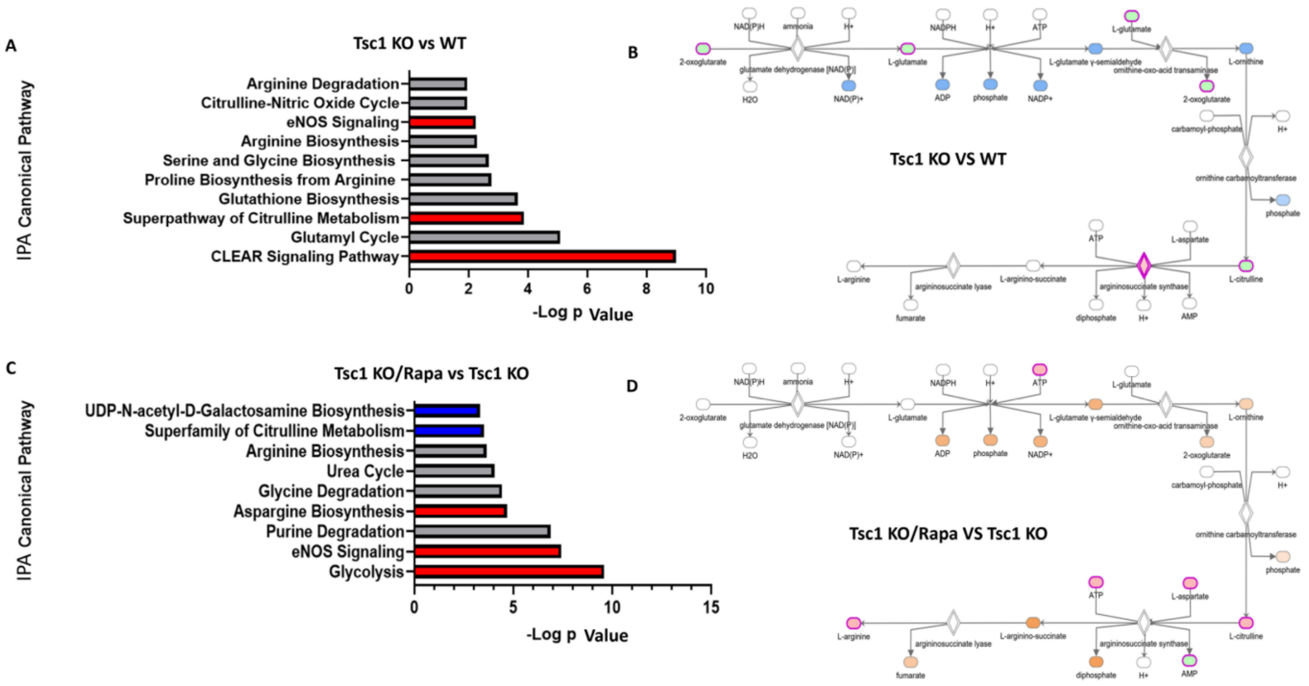

Figure S2: <u>Potential Canonical Pathways and significantly differentially expressed genes and metabolites affected by *Tsc1*K O and treatment with rapamycin, Related to Figure 2.</u>

QIAGEN IPA® of canonical pathways that may be affected (log P-value>1.3) by the significantly differentially expressed genes and metabolites of *Tsc1* KO vs. WT (**A**) and *Tsc1* KO vs. *Tsc1* KO mice treated with rapamycin (**C**). QIAGEN IPA® z-scores larger than 2 or smaller than -2 are considered significantly predicted to be up (red bars) or down (blue bars)- regulated. Gray bars are for canonical pathways with no calculated QIAGEN IPA® z-scores. Scheme of QIAGEN IPA® of arginine Biosynthesis IV canonical pathways for *Tsc1* KO vs. WT (**B**) and *Tsc1* KO treated with rapamycin vs. *Tsc1* KO treated with vehicle (**D**). Experimentally significantly differentially expressed genes and metabolites are highlighted in magenta and colored in pink (upregulated) and green (downregulated). Predicted upregulated and down-regulated genes and metabolites, using the QIAGEN IPA® MAP (Molecule Activity Predictor) tool, are depicted in orange and blue, respectively. Ellipse and rhombus represent chemical and kinase molecules, respectively.

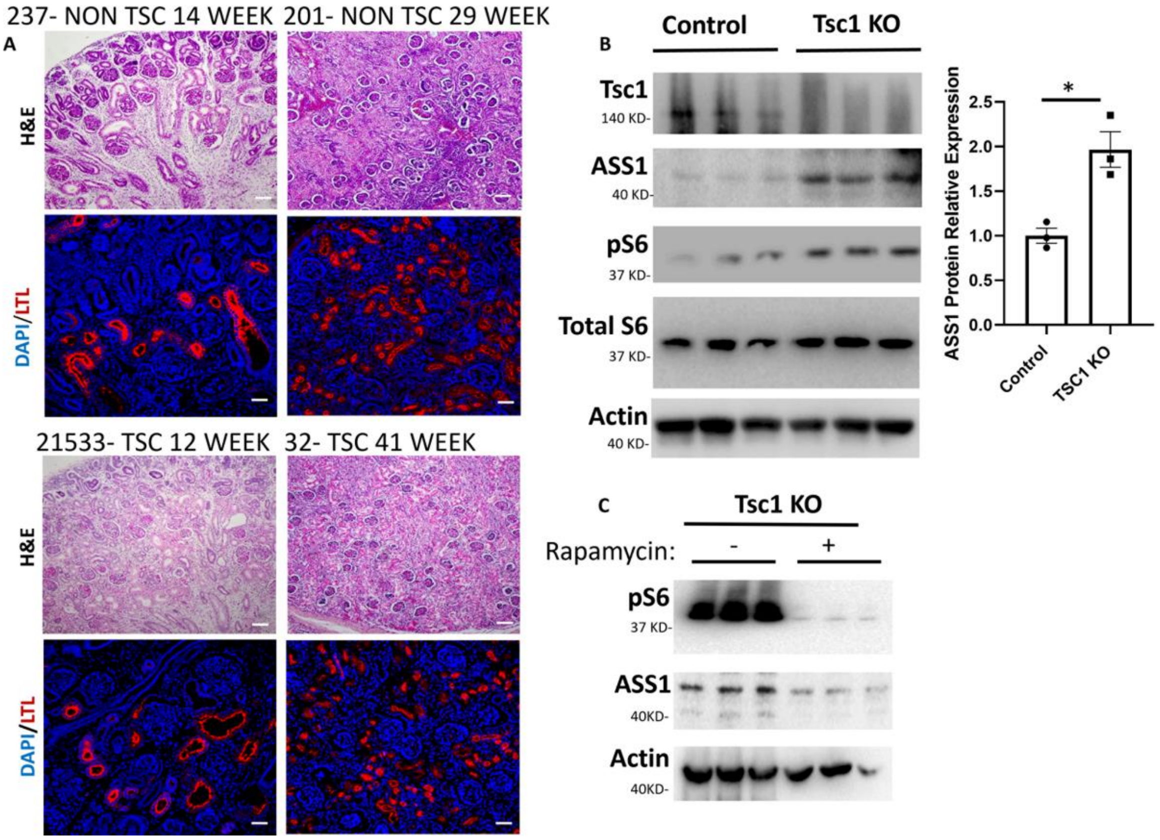

Figure S3: *Tsc1* KO induces ASS1 expression in a mTORC1-dependent manner, Related to Figure 3.

Embryonic kidney sections from human TSC and non-TSC patients in early and late matched pregnancy were H&E stained or immunostained for Lotus Tetragonolobus Lectin (LTL) as a specific marker for PTCs (**A**), showing no difference in the immunostaining. (**B**) Western blot for TSC1, pS6 (a marker for mTORC1 activation), Total S6, Actin, and ASS1 in extracts obtained from control or *Tsc1* KO-induced HK2 cells (n=3 in each group, biological replicates). On the right panel, ASS1 relative protein expression is indicated. *$p < 0.05$. (**C**) Western blot analysis for ASS1, pS6, and actin protein expression in *Tsc1* KO HK2 either incubated with vehicle or 50 nM rapamycin for 24 hours (n=3 in each group, biological replicates).

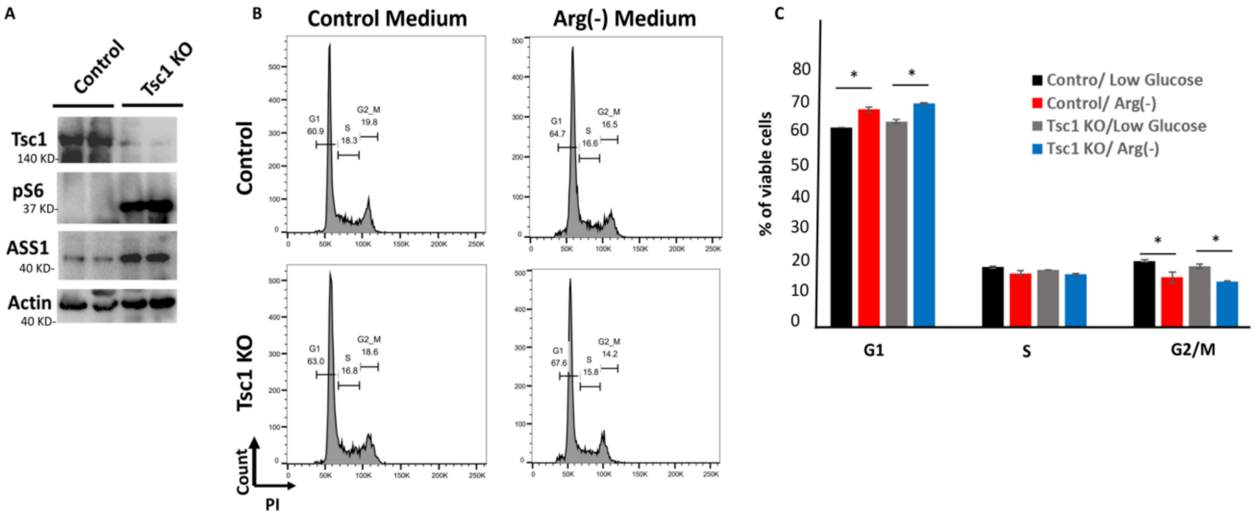

Figure S4: *Tsc1* KO induces ASS1 overexpression in HEK293 cells, Related to Figure 5.

HEK293 cells were cultured in DMEM medium and infected with ASS1/CRISPR or control-containing lentivirus particles. The cells were puromycin and GFP^{+} based selected. (**A**) Western blot for TSC1, pS6 (a marker for mTORC1 activation), Actin, and ASS1 in extracts obtained from control or *Tsc1* KO-induced HEK293 cells (n=2, biological replicates). (**B**) Control and *Tsc1* KO HEK293 cells were incubated with either control or arginine-free medium for 10 days. Cells were harvested and fixed, and the cell cycle was monitored by propidium iodide flow cytometry-based analysis. (**C**) Quantification as in B (n=3, biological replicates). *p<0.05.

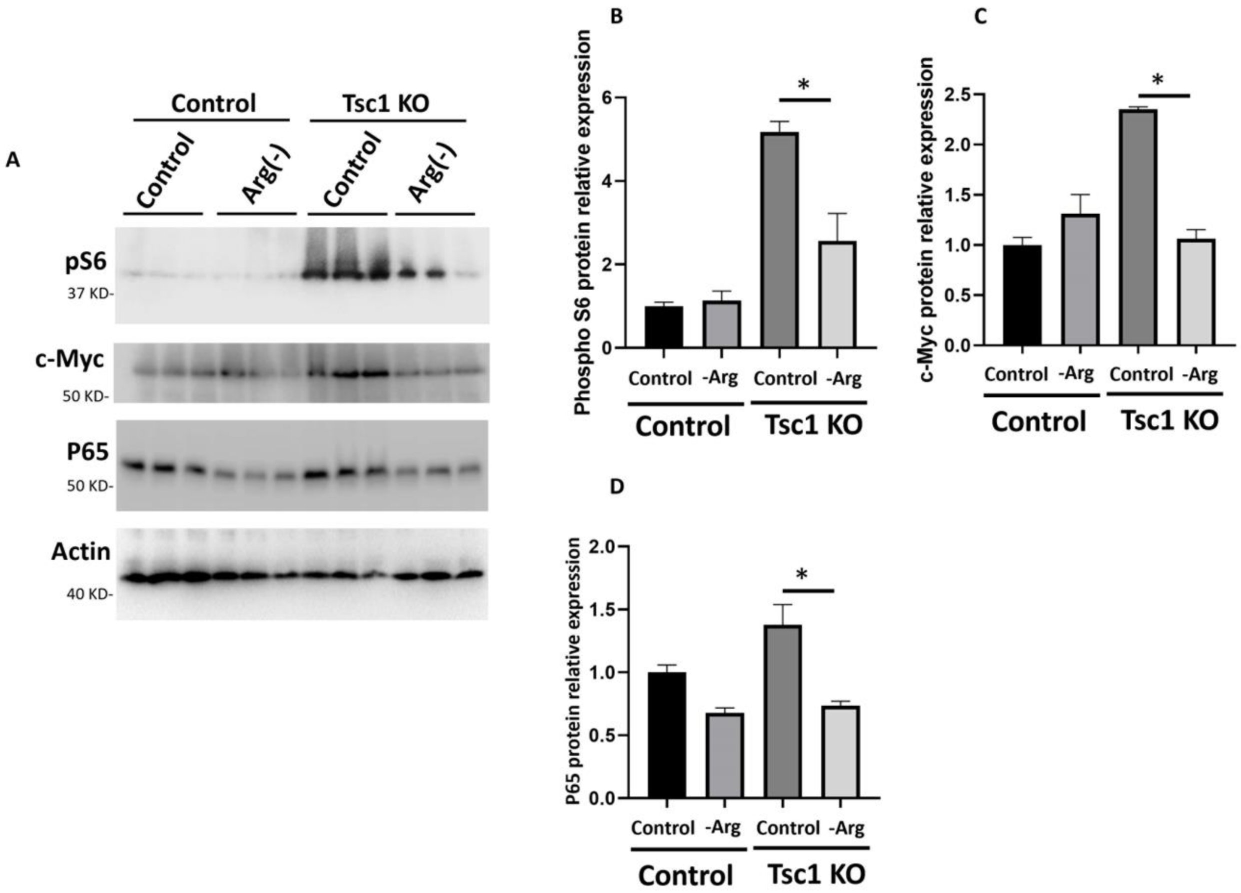

Figure S5: <u>Arginine depletion *in vitro* attenuates TSC-associated signaling in HEK293 cells, Related to Figure 5.</u>
Control and *Tsc1* KO HEK293 cells were incubated with either control or arginine-free medium for 10 days. (**A**) Cells were harvested, and the expression pS6, c-Myc, P65, and actin were monitored by western blot analysis, (n=3 in each group, biological replicates). (**B-D**) Quantification of the indicated proteins as in A (n=3, biological replicates). *p<0.05.

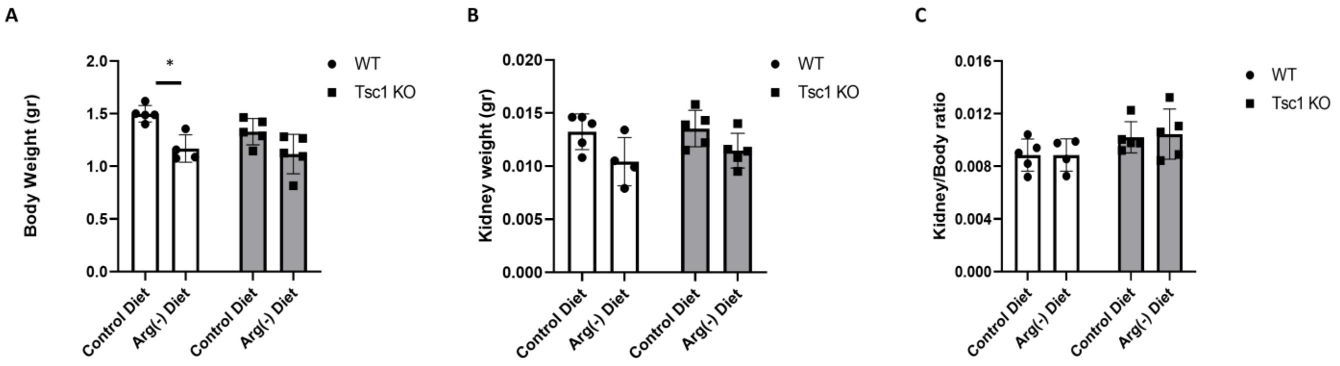

Figure S6: **<u>An arginine-deficient diet does not induce kidney and/or body weight changes in newborns, Related to Figure 6.</u>**

(**A**) Total body weight of WT and *Tsc1* KO pups from *Tsc1* $^{fl/fl}$ mothers fed with either control or arginine deficient diets at P0 (n=4, three litters, biological replicates) was measured. (**B**) Weight of two kidneys of each pup as in (A) at P0. (**C**) Weight of the two kidneys as in (B) compared with total body weight at P0. *p<0.05.

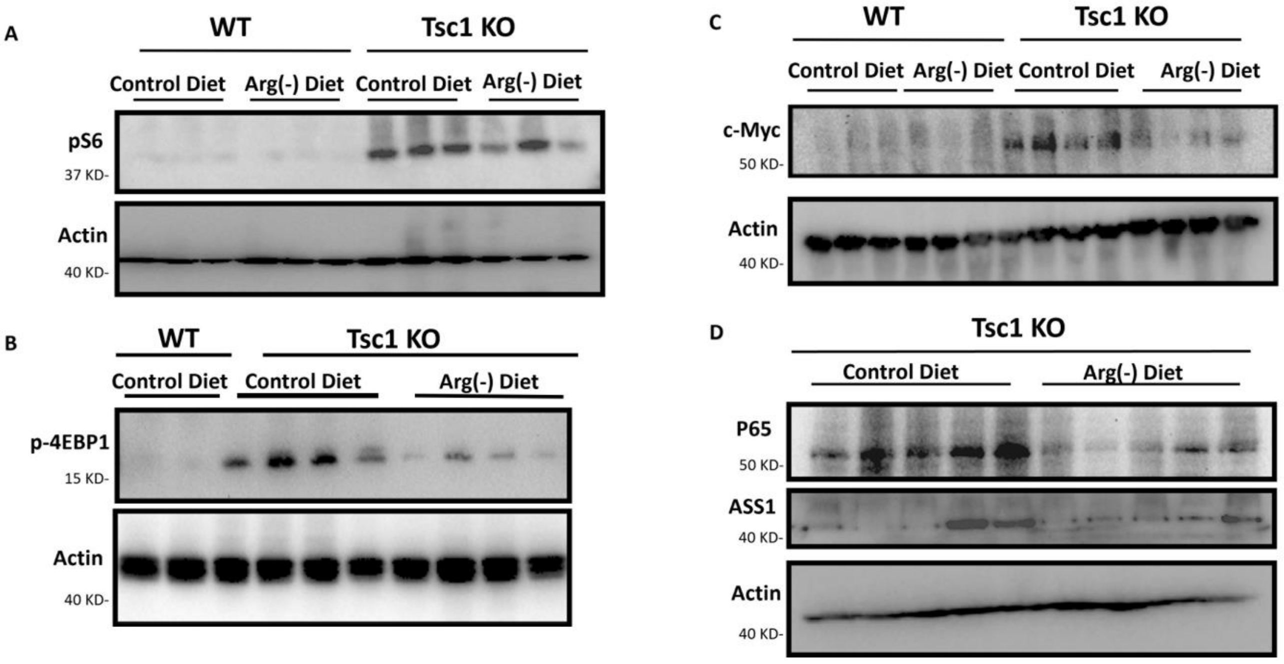

Figure S7: <u>Arginine depletion attenuates mTOR signaling and c-Myc and NF-κB P65 subunit protein expression *in vivo* in *Tsc1* KO kidneys, Related to Figures 6 and 7.</u>

(**A**) Western blot for pS6 and actin protein expression in kidney extracts from WT and *Tsc1* KO pups of *Tsc1* $^{fl/fl}$ mothers fed either a control or an arginine deficient diet, at P0, (n=3 each, biological replicates). (**B**) Western blot for p-4EBP1 and actin protein expression using kidney extracts from WT (n=2 each, biological replicates) and *Tsc1* KO (n= 4 each, biological replicates) pups of *Tsc1* $^{fl/fl}$ mothers fed either control or arginine depleted diets at P0. (**C**) Western blot for c-Myc and β-actin protein expression using kidney extracts from WT (n=3 each, biological replicates) and *Tsc1* KO (n= 4 each, biological replicates) pups of *Tsc1* $^{fl/fl}$ mothers fed either a control or arginine-depleted diet at P0. (**D**) P65, ASS1, and β-actin protein expression using kidney extracts from *Tsc1* KO (n= 5 each, biological replicates) pups of *Tsc1* $^{fl/fl}$ mothers fed either control or arginine-depleted diet, at P0.

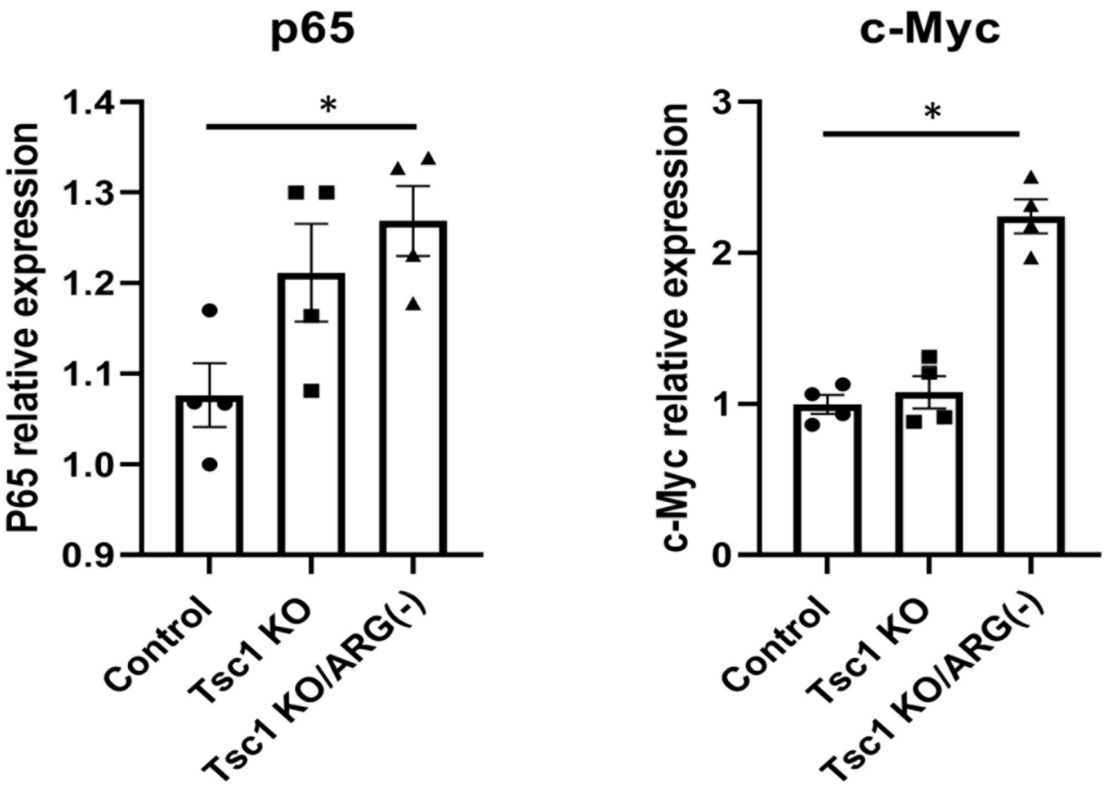

Figure S8: **<u>Arginine depletion does not affect c-Myc and P65 gene expression, Related to Figure 7.</u>** RNA from WT and *Tsc*1 KO kidneys fed either a control or arginine-depleted diet as indicated were extracted. The relative expression of P65 and c-Myc was monitored and analyzed against β-actin RNA expression by qRT-PCR. WT pups fed a control diet (n = 4, biological replicates), *Tsc1* KO pups fed a control (n = 4, biological replicates), and arginine deficient diet (n = 4, biological replicates), $^*p < 0.05$.

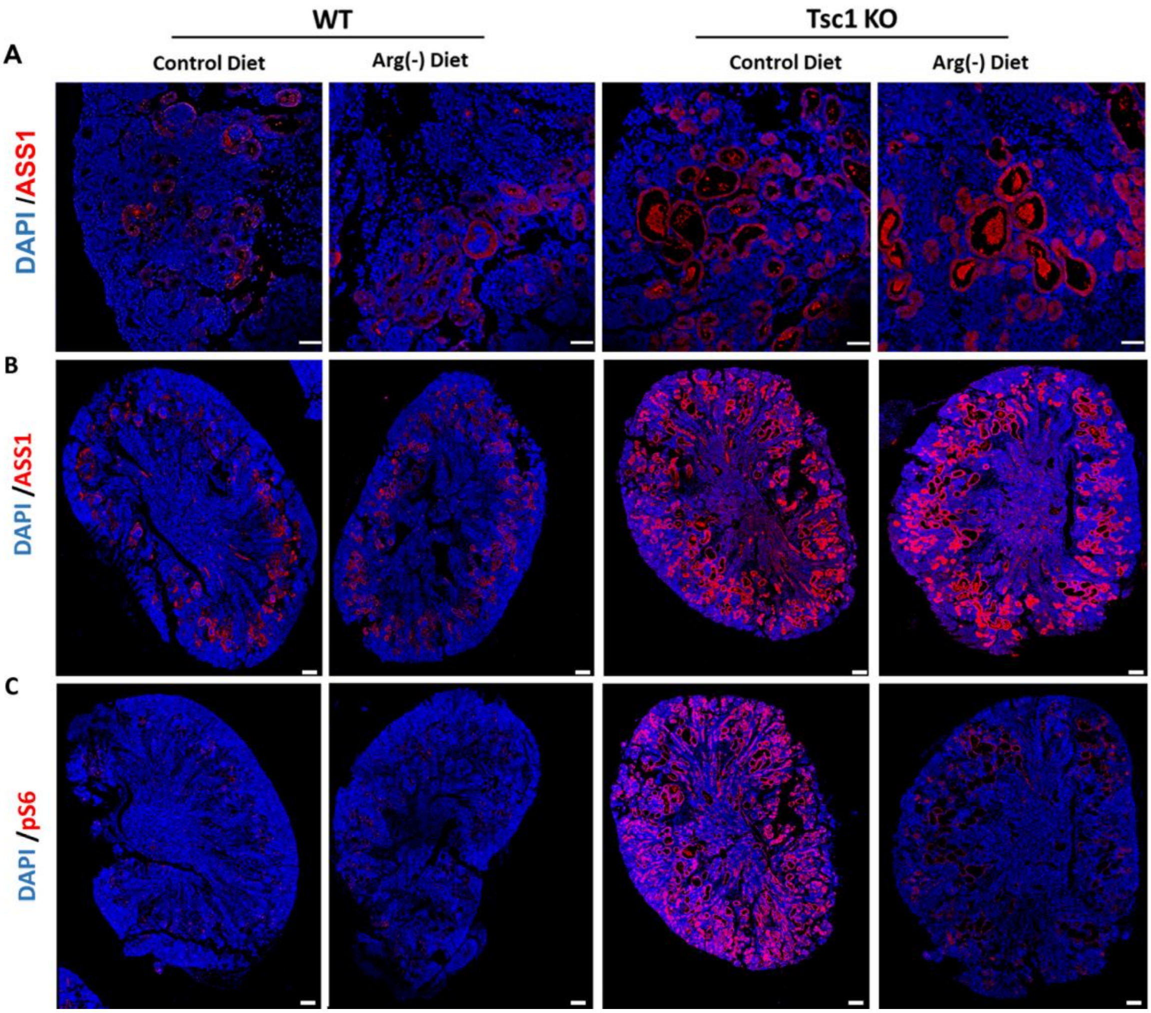

Figure S9: **<u>An arginine-depleted diet does not affect ASS1 expression, Related to Figures 6 and 7.</u>** (**A**) Kidney sections from WT and *Tsc1* KO P0 pups from *Tsc1* $^{fl/fl}$ mothers fed either a control or an arginine-deficient diet were immunostained for ASS1. Scale bar: 50 μm. WT pups were fed a control diet (n = 4, biological replicates) and an arginine-deficient diet (n = 3, biological replicates). *Tsc1* KO pups were fed a control diet (n = 4, biological replicates) and an arginine-deficient diet (n = 4, biological replicates). Sections, as in A, were immunostained for ASS1 (**B**) or pS6 as a marker for mTORC1 activation (**C**). WT pups fed a control diet (n = 3, biological replicates), *Tsc1* KO pups fed a control diet (n = 3, biological replicates), or Arginine depleted diet (n = 3, biological replicates), Scale bar = 500 μm.

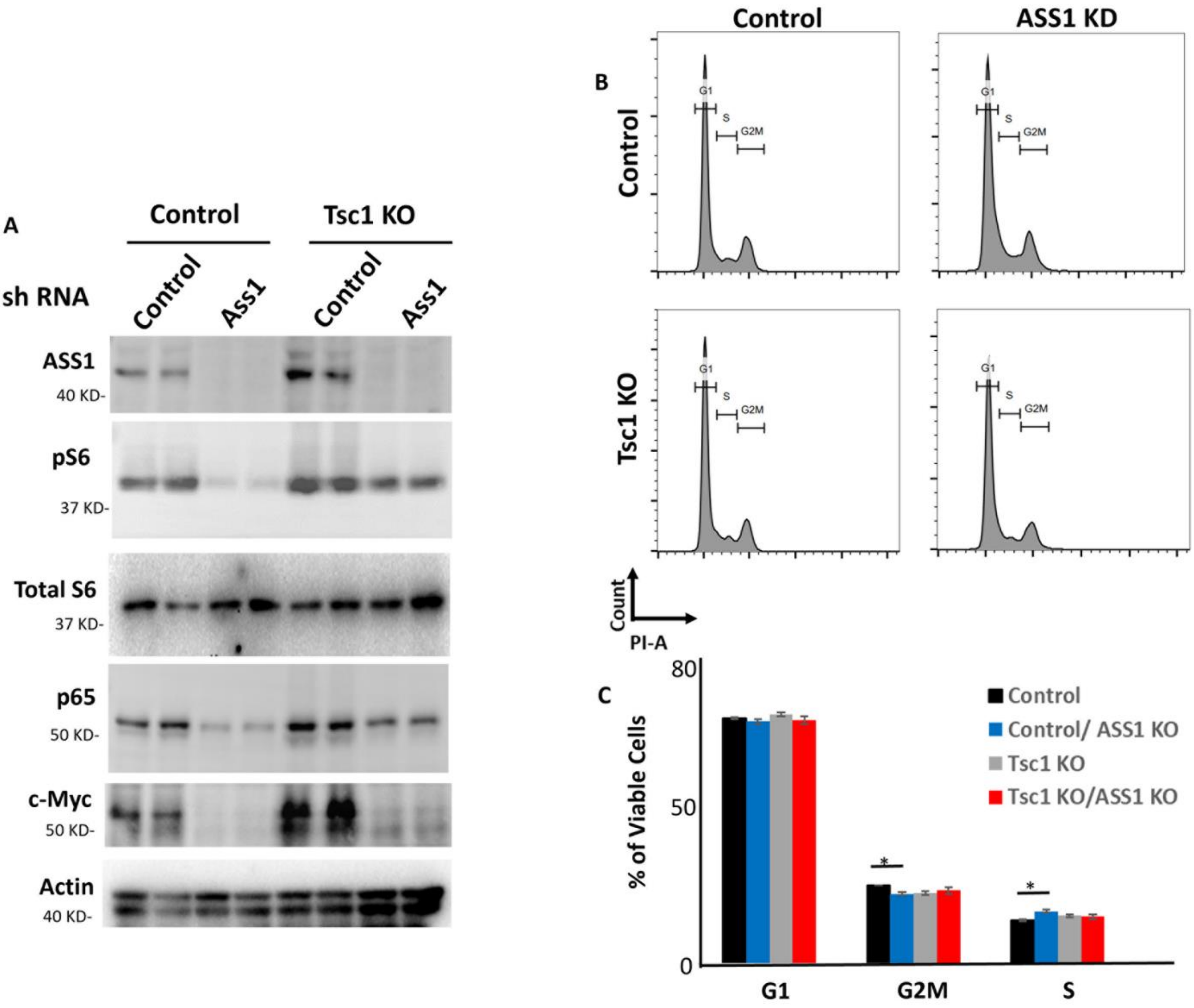

Figure S10: **<u>ASS1 Knock down attenuates TSC associated cell signaling in HK2 cells, Related to Figure 7.</u>**
(**A**) Control and *Tsc1* KO HK2 cells were infected with lentivirus particles containing either control or ASS1-targeted shRNA. After neomycin-based selection, the relative protein expression for ASS1, total and ribosomal protein pS6, P65, c-Myc and actin were evaluated by WB, (n=2, biological replicates). (**B**) Control and *Tsc1* KO HK2 cells, as in A, were harvested and fixed, and the cell cycle was monitored by propidium iodide flow cytometry-based analysis. (**C**) Quantification as in B (n=3, biological replicates). *$p<0.05$.
